# HFGuidedDesign: *de novo* design of cyclic peptide binders *via* structure-guided discrete diffusion

**DOI:** 10.1039/d6sc02631a

**Published:** 2026-06-30

**Authors:** Haomeng Hu, Renjie Zhu, Ning Zhu, Chengyun Zhang, Tianfeng Shang, Chongyang Li, Jingjing Guo, Xudong Wang, Hongliang Duan

**Affiliations:** a College of Pharmaceutical Sciences, Zhejiang University of Technology Hangzhou 310014 China xdwang2019@zjut.edu.cn; b Faculty of Applied Sciences, Macao Polytechnic University Macao 999078 China hduan@mpu.edu.mo; c AI Department, Shenzhen Highslab Therapeutics. Inc Shenzhen 518000 China

## Abstract

Cyclic peptides are promising scaffolds for targeting protein surfaces due to their unique structural and functional advantages. However, the limited availability of cyclic peptide–protein complex structures severely restricts the design of target-specific cyclic peptides. Here, we introduce HFGuidedDesign, a *de novo* cyclic peptide design framework that integrates a discrete diffusion model with external structure guidance. By incorporating the high-accuracy complex structure predictor HighFold, the framework performs real-time structural evaluation during reverse diffusion sampling and dynamically steers sequence generation toward cyclic peptides with favorable structural plausibility and binding potential. The discrete diffusion model is trained using a two-stage strategy, including pre-training on peptide monomers and fine-tuning on peptide–protein complex structures. In design tasks targeting two distinct proteins, we evaluate two classical cyclization strategies—head-to-tail and disulfide bond cyclization. The resulting cyclic peptides achieved sequence design success rates of 75% and 66.7% for the two targets, demonstrating the effectiveness and generalizability of the framework. This study establishes an innovative and scalable computational framework for sequence-based cyclic peptide design, facilitating the development of peptide-based ligands for protein targeting.

## Abstract 2

HFGuidedDesign integrates discrete diffusion with HighFold-guided structural feedback to generate cyclic peptide binders from protein sequences, followed by synthesis and SPR validation.

## Graphical abstract

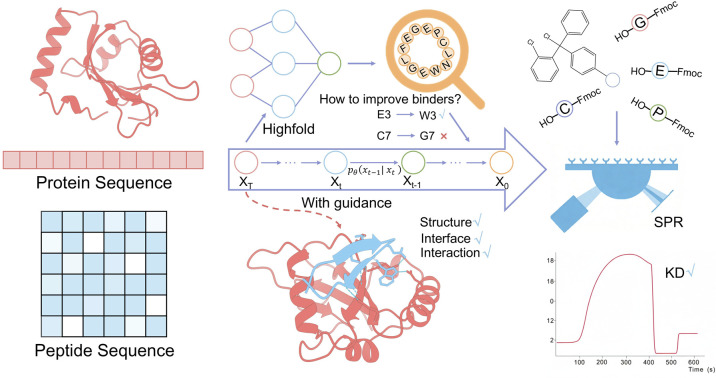

## Introduction

Proteins carry out their biological functions by engaging in specific interactions with other molecules, including nucleic acids, small molecules, and other proteins. Modulating protein–ligand interactions has therefore emerged as an important strategy for therapeutic intervention across a wide range of diseases. However, many functionally relevant protein surfaces remain challenging to target with conventional drug modalities. Large biologics, such as monoclonal antibodies and protein scaffolds, offer high affinity and specificity but often fail to reach intracellular targets because of poor membrane permeability. In contrast, small-molecule drugs readily penetrate cells but typically struggle to engage extended or shallow protein surfaces that lack well-defined binding pockets. Peptides, as a class of compounds between proteins and small molecules, combine the structural versatility and high binding affinity of proteins with the potential cell permeability of small molecules, making them attractive candidates for targeting so-called undruggable protein surfaces.^1–3^ Among them, cyclic peptides, due to their unique cyclic structure, exhibit significantly enhanced conformational stability, superior resistance to enzymatic degradation, higher binding affinity and target specificity, rendering them particularly attractive scaffolds for protein targeting.^4–6^

Despite their considerable therapeutic potential, the rational design of cyclic peptides remains challenging for several reasons. First, cyclic peptides possess an exceptionally vast sequence space coupled with high conformational diversity. Second, different cyclization modalities impose markedly distinct structural constraints.^7^ Traditionally, the discovery of cyclic peptide-based therapeutics has relied primarily on natural product mining or display technologies, such as phage display and mRNA display, for targeted screening of ultra-large peptide libraries.^8,9^ While these approaches have successfully identified high-affinity binders, they are universally constrained by several limitations: natural products are often scarce, synthetically challenging, and yield lead compounds with poor stability and low mutational tolerance.^10^ Although display-based methods have the advantage of high throughput, it is time-consuming, costly, and can only cover a small part of the cyclic peptide chemistry and structural space.^11^

A variety of computational approaches have been developed for cyclic peptide design. However, most existing pipelines adopt a two-stage strategy to generate cyclic peptide binders for specific targets. Specifically, researchers commonly employ structure diffusion-based design methods^12,13^ or AlphaFold-based backpropagation strategies^14–16^ to construct cyclic peptide backbones that satisfy spatial and geometric constraints, followed by sequence design using ProteinMPNN^17^ and its iterative variants.^18^ Despite its effectiveness, this two-step workflow inherently decouples conformational modeling from sequence design. This leads to a stage-wise, funnel-like design paradigm in which modeling and prediction errors progressively propagate and accumulate across successive steps. This error accumulation markedly compresses the overall success rate, leaving only ∼4–7% of ProteinMPNN-generated sequences as predicted by AlphaFold2 (ref. 19) and other structure prediction tools to fold into high-confidence cyclic conformations.^20^ Moreover, such error propagation not only reduces the efficiency of sequence screening but may also cause potentially active scaffolds to receive low-confidence sequences during inverse folding, leading to their premature elimination before experimental validation. Recently, several studies have explored end-to-end peptide sequence design using protein language models. For example, PepMLM^21^ fine-tunes ESM-2 (ref. 22) on protein–peptide complex data to directly generate linear peptide binders for specific protein targets. However, extending such approaches to cyclic peptide design remains challenging. First, the vast majority of complexes in the current PDB involve linear peptide–protein interactions, resulting in a scarcity of high-quality and diverse cyclic peptide–protein complex data for effective model training. Second, the input representation of ESM-2 is inherently limited to linear sequences, making it difficult to adequately capture the spatial constraints and long-range dependencies imposed by cyclic topologies.

In this study, we present HFGuidedDesign, a novel framework for cyclic peptide binder design based on a sequence-level discrete diffusion model augmented with external structural guidance. We train and fine-tune the discrete diffusion model on extensive datasets of peptide monomers and peptide–protein complexes, enabling it to generate peptide sequences conditioned solely on the target protein sequence. To enhance the structural plausibility and binding propensity of the generated sequences, we incorporate our proposed HighFold^23^ as an external structural guidance module. HighFold is a high-accuracy predictor of cyclic peptide–protein complexes that is highly sensitive to subtle mutations at key binding residues and can precisely capture how sequence variations affect complex stability and binding conformations. HighFold performs real-time structural evaluation of intermediate sequences during the reverse diffusion sampling process. Guidance signals are constructed based on metrics such as interface accuracy and local structural confidence, which are then used to dynamically adjust the denoising distribution, thereby steering the discrete diffusion model to generate cyclic peptide sequence ensembles with both structural plausibility and high binding potential. We evaluate HFGuidedDesign on two distinct protein targets. For both targets, the framework successfully generated cyclic peptide sequences with measurable binding affinity, demonstrating its effectiveness and generalizability for target-specific cyclic peptide design.

## Methods

### Dataset construction for HFGuidedDesign

We employ a two-stage training strategy for the diffusion module in the HFGuidedDesign model. In the first stage, the model is pretrained on peptide monomer sequences to learn basic sequence patterns and amino acid composition properties. Peptide sequences are curated from the UniRef50 (ref. 24) database by selecting sequences with lengths not exceeding 50 residues and containing only the 20 canonical amino acids, yielding approximately 1.94 million peptide sequences for pretraining the peptide monomer diffusion model. In the second stage, we fine-tune the model on peptide–protein complex data to enable learning of interaction patterns between peptides and their target proteins. We integrate two datasets for this purpose: PPIKB,^25^ a comprehensive database containing protein–peptide complexes together with their corresponding binding affinity data, and another dataset used to train the PepMLM model. To ensure data quality and training efficiency, we apply the following filtering criteria: target protein length ≤500 residues and peptide length ≤50 residues. After removing duplicate entries between the two datasets and excluding complexes containing non-canonical amino acids, we retain 9511 high-quality peptide–protein complex samples for fine-tuning the pretrained peptide monomer diffusion model.

### Model architecture of HFGuidedDesign

**Fig. 1:**
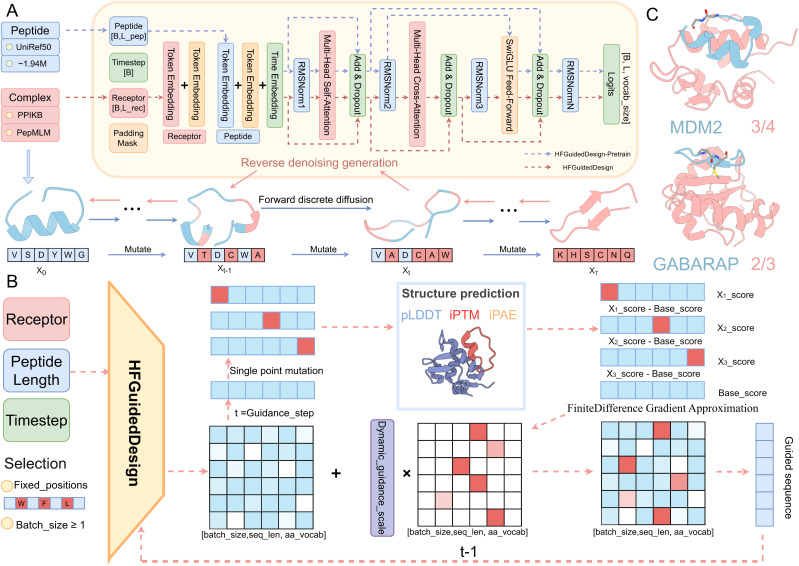
Overall architecture and structure-guided generation framework of HFGuidedDesign. (A) Illustrates the training pipeline of HFGuidedDesign, including the forward diffusion process and the reverse denoising generation process, as well as the pretraining and fine-tuning stages using peptide monomer datasets and peptide–protein complex datasets, respectively. (B) Shows the structure-guided reverse diffusion process, where a structure prediction model evaluates intermediate peptide sequences and provides guidance signals that steer peptide generation toward high structural confidence and binding potential. (C) Design success rates of cyclic peptide binders for the MDM2 and GABARAP targets achieved by HFGuidedDesign.

HFGuidedDesign builds on the discrete denoising diffusion probabilistic model (D3PM) framework^26,27^ to generate cyclic peptide sequences in a discrete space of canonical amino acids, as illustrated in Fig. 1. The model defines a forward noising process and a reverse denoising process, enabling the progressive reconstruction of cyclic peptide sequences conditioned on a target protein sequence. The model aims to generate ensembles of cyclic peptide binders with potential binding activity given a target protein sequence. The overall architecture of HFGuidedDesign consists of two core components: a discrete denoising diffusion module and a structure-guided module. The former performs diffusion and denoising operations in discrete sequence space to iteratively generate cyclic peptide sequences. The latter utilizes structural information of the complex formed between the target protein and the currently generated cyclic peptide as conditional input and imposes structural and binding preference guidance on the generation process, thereby enhancing the structural plausibility and binding potential of the generated cyclic peptide sequences.

### Discrete denoising diffusion module

#### Forward diffusion process

During the forward diffusion stage, at each time step *t* = 1, 2, …, *T*, noise is progressively added to the initial peptide sequence using a time-dependent stochastic transition matrix *Q*_*t*_. Specifically, each amino acid remains unchanged with probability 1 − *β*_*t*_, and is replaced with a uniformly sampled amino acid from the vocabulary with probability *β*_*t*_. By sequentially applying the transition matrices *Q*_*t*_ across time steps, the original sequence distribution is gradually transformed into an approximately uniform noise distribution, thereby forming a forward Markov chain. For peptide–protein complex data, we apply noise exclusively to the ligand (peptide) sequence while keeping the receptor (target protein) sequence fixed. This design preserves the integrity and stability of the conditioning information and avoids introducing unnecessary noise into the receptor sequence. Specifically, the transition matrix at time step is defined as:
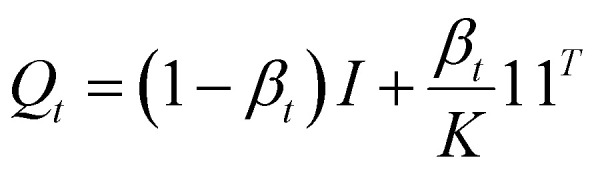
where *K* denotes the vocabulary size, *I* is the identity matrix, and 1 is an all-ones vector.

The cumulative transition matrix is given by 
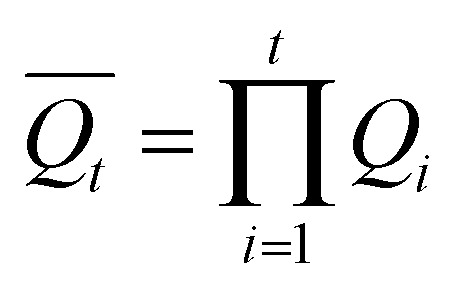
 which characterizes the direct transition from the clean sequence *X*_0_ to the noisy sequence. This formulation enables efficient sampling of noisy sequences *via*
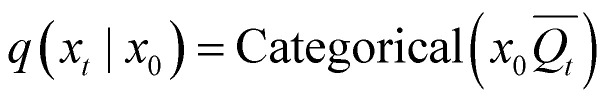


To ensure smooth and stable noise evolution throughout the diffusion process, we adopt the noise schedule proposed by Sohl-Dickstein,^28^ defined as
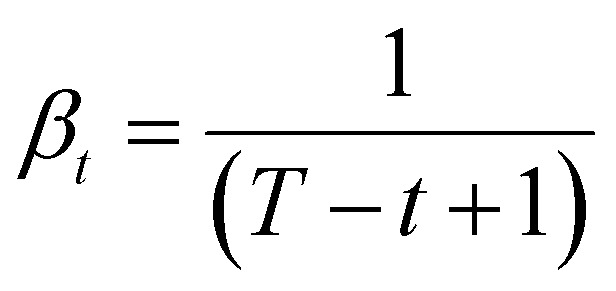


This schedule balances noise intensity across diffusion steps, thereby improving stability and reconstruction quality during the reverse denoising process.

#### Reverse denoising process

During the reverse diffusion stage, HFGuidedDesign employs a conditional denoising network based on a Transformer architecture^29^ to model the reverse transition distribution *p*_*θ*_(*x*_*t*−1_|*x*_*t*_,*r*), at each time step *t*, progressively recovering the original peptide sequence *x*_0_. Here, *r* denotes the sequence-level features of the target protein. The primary objective of the denoising network is to learn conditional distributions across different noise levels, enabling the stepwise reconstruction of clean cyclic peptide sequences. At the input level, the denoising network jointly receives three types of information: (1) the representation of the current noisy peptide sequence *x*_*t*_, (2) embedded features of the target protein sequence, and (3) an embedding of the current diffusion time step *t*, which explicitly encodes the noise level and diffusion stage. The transformer architecture incorporates multi-head self-attention and cross-attention mechanisms to model intra-peptide residue dependencies and peptide–protein interaction patterns, respectively. Through cross-attention layers, peptide representations are deeply integrated with receptor sequence embeddings, progressively aggregating cross-sequence contextual information to guide the generation of peptide sequences with binding potential. Because early diffusion stages favor global structural coherence while later stages demand fine-grained local detail refinement, we further enhance temporal awareness by introducing Feature-wise Linear Modulation (FiLM)^30^ into the normalization layers of the transformer. Specifically, for an activation tensor *h*, the FiLM module generates time-dependent scaling and shifting parameters *γ*_*t*_ and *β*_*t*_ from the time-step embedding and applies linear modulation asFiLM(*h*,*t*) = γ_*t*_ ⊙*h* + *β*_t_where ⊙ denotes element-wise multiplication. This mechanism enables the model to dynamically adapt feature distributions according to the current noise level, improving robustness during high-noise stages and enhancing structural detail reconstruction during low-noise stages.

#### Training loss

To effectively train HFGuidedDesign, we adopt a hybrid loss function that combines a variational lower bound (VLB) loss 
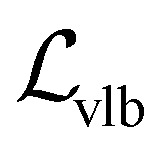
 and a cross-entropy loss 
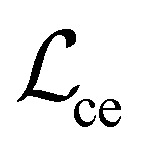
, ensuring both generation quality and training stability. The total loss is defined as
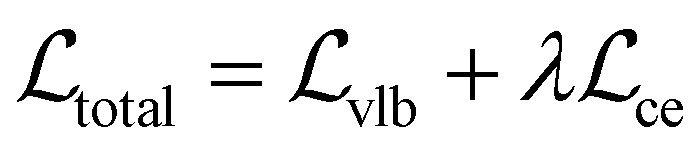
where *λ* is a hyperparameter that balances the two terms.

The VLB loss 
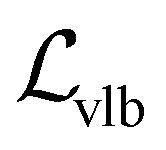
 is derived from the evidence lower bound (ELBO) of the discrete diffusion process and is computed across diffusion time steps. At intermediate steps (1 < *t* < *T*), the VLB term minimizes the Kullback–Leibler divergence between the true posterior distribution *q*(*x*_*t*−1_|*x*_*t*_,*x*_0_) and the model-predicted distribution *p*_*θ*_(*x*_*t*−1_|*x*_*t*_). At the final diffusion step (*t* = *T*), the loss reduces to a prior-matching term that enforces the fully noised sequence to follow a uniform prior distribution. At the initial reconstruction step (*t* = 0), the VLB loss becomes equivalent to a negative log-likelihood term, directly optimizing the model's ability to reconstruct the original sequence. Formally, the VLB loss is given by
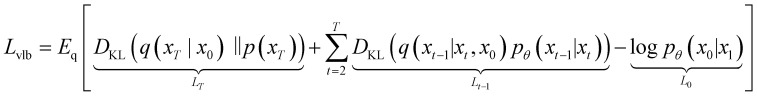
Here, *D*_KL_corresponds to the prior-matching term. The distribution *q*(*x*_*t*−1_|*x*_*t*_,*x*_0_) denotes the true posterior defined by the discrete diffusion process, whereas *p*_*θ*_(*x*_*t*−1_|*x*_*t*_) represents the model-induced approximate posterior derived from the prediction of *x*_0_.

The auxiliary cross-entropy loss 
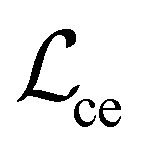
 directly measures the discrepancy between the predicted denoised sequence *x*_0_ and the ground-truth sequence *x*_0_, and is defined as
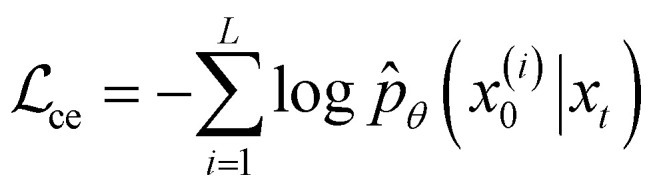


This auxiliary objective effectively mitigates gradient vanishing and training instability associated with optimizing the VLB loss alone, particularly during early training stages.

### External diffusion guidance generation module

Although the diffusion model is trained on peptide–protein complex data, the relatively low proportion of cyclic peptide–protein complexes in the training set limits its ability to reliably design high-affinity cyclic peptide binders for specific targets. Moreover, the diffusion-based generation process lacks explicit structural or functional constraints, making it difficult to directly guarantee the three-dimensional structural plausibility of generated peptides or their binding propensity toward the target protein.

To overcome these limitations, HFGuidedDesign introduces an adaptive external diffusion guidance module that dynamically injects structure-aware feedback signals at specific timesteps during the reverse diffusion process, thereby steering the generation trajectory toward regions of amino acid sequence space that simultaneously exhibit high structural plausibility and strong target-binding propensity.

The core of the guidance strategy combines gradient estimation of a structure-based objective and directed exploration in discrete sequence space, thereby actively guiding the peptide sequence to evolve towards a direction with higher structural quality and stronger binding ability to the target. Specifically, at each predefined guidance time step *t*_g_, for the current noisy peptide sequence *x*_*t*_, the guidance module first invokes the structure prediction model HighFold to predict the complex formed by *x*_*t*_ and the input target protein sequence.

To comprehensively evaluate the quality of the generated cyclic peptide sequences, we assess three complementary aspects critical to peptide–protein design: interface quality, peptide structural confidence, and predicted positional accuracy at the interface. Accordingly, three structure-prediction-derived metrics (iPTM, pLDDT_peptide_, and iPAE) are extracted from the HighFold predictions and combined into a composite structural score. Specifically, iPTM measures the predicted accuracy of the peptide–protein interface, pLDDT_peptide_ reflects the local structural confidence of the peptide chain, and iPAE captures the predicted alignment error between interfacial residue pairs. These metrics are widely adopted as reliable criteria for ranking and filtering candidate sequences in peptide design tasks. Based on these outputs, a composite structural score *S*_base_ is defined as:*S*_base_ = *w*_1_ × iPTM + *w*_2_ × pLDDT_peptide_ + *w*_3_ × (1 − iPAE)

Subsequently, the model employs a sparse and adaptive candidate mutation strategy to generate a set of informative single-point mutant sequences. At the position level, only a subset of residue positions with higher optimization potential is selected at each guidance step. The number of selected positions is adaptively adjusted according to the gap between the current composite structural score and the target score, with more positions selected for broader exploration when the gap is large and fewer positions selected for local refinement when the gap is small. Candidate positions are prioritized according to model uncertainty, estimated from the entropy of the predicted amino-acid distribution and the confidence gap between the top-ranked amino-acid probabilities. At the amino-acid-substitution level, candidate substitutions are selected using a UCB1-based strategy^31^ which maintains historical mutation statistics for each position-amino-acid pair and balances exploration of insufficiently sampled substitutions with exploitation of previously beneficial mutations. In this way, the mutation search is restricted to a compact set of promising candidate substitutions while avoiding exhaustive evaluation of the full mutation space.

All candidate mutant sequences are then batch-evaluated using HighFold to obtain their corresponding structural scores *S*_seq_. Based on the difference between the baseline score *S*_(*x*_*t*_)_ and the mutant score 
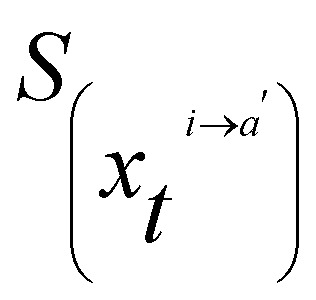
, the guidance module constructs a position-amino-acid-level gradient signal ∇*S* using a finite-difference approximation in discrete sequence space:
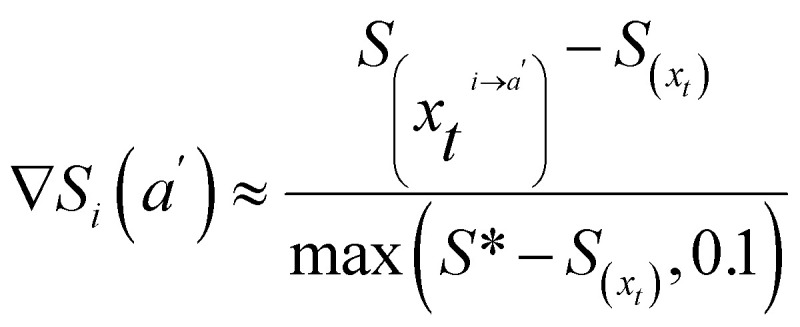
where S* denotes the predefined maximum structural score.

The resulting gradient signal is incorporated into the denoising distribution in a weighted manner to achieve directed correction of the sampling trajectory. Concretely, at each guidance time step *t*_g_, the model first computes the original conditional denoising distribution *p*_*θ*_(*x*_*t*−1_|*x*_*t*_,*r*), and then augments its log-probability with the guidance term *w*_g_ × ∇*S*:log *p*_guided_ (*x*_*t*−1_|*x*_*t*_,*r*) = log(*p*_*θ*_(*x*_*t*−1_|*x*_*t*_,*r*)) + *w*_g_ × ∇*S*where *w*_g_ is an adaptive guidance strength coefficient that modulates the influence of structural guidance across diffusion stages. The guided distribution *p*_guided_ is obtained *via* softmax normalization and used to sample the next-step sequence *x*_*t*−1_. This guided sampling strategy preserves the stochasticity and diversity of the diffusion process while effectively steering generation toward high-scoring regions of sequence space, resulting in cyclic peptide sequences with enhanced structural plausibility and target-binding potential.

### Cyclic peptide synthesis and purification

All cyclic peptides were synthesized using standard Fmoc-based solid-phase peptide synthesis (Fmoc-SPPS) on 2-chlorotrityl chloride (2-CTC) resin.^32^ The C-terminal amino acid was first coupled to the resin in the presence of *N*,*N*-diisopropylethylamine (DIEA). Fmoc deprotection was carried out using 20% piperidine in DMF, followed by stepwise chain elongation under DIC/HOBt coupling conditions until the full linear peptide sequence was assembled. For amide-bond cyclization, fully protected linear peptides were cleaved from the resin and subjected to intramolecular head-to-tail cyclization under DIC/HOBt conditions. Side-chain protecting groups were subsequently removed using a TFA-based cleavage cocktail to yield the final cyclic peptides. All cyclic peptides were purified by preparative reversed-phase high-performance liquid chromatography (RP-HPLC) using a 0.1% TFA in water/acetonitrile solvent system. Final peptide purities exceeded 95%, and molecular weights were confirmed by LC-MS (SHIMADZU LCMS-2020).

### Affinity measurement with SPR

To evaluate the binding affinity of the designed cyclic peptides toward the target protein, surface plasmon resonance (SPR) measurements were performed using a Biacore S200 instrument.^33^ All experiments were conducted at 25 °C in PBST buffer (1× PBS supplemented with 0.05% Tween-20) containing 0.5% (v/v) DMSO. A 0–1% DMSO gradient was applied for solvent correction. The target protein was immobilized on a CM5 sensor chip *via* standard amine coupling. The chip surface was cleaned with NaOH/SDS, activated using EDC/NHS, and the target protein (10 µg mL^−1^ in pH 4.0 sodium acetate buffer) was injected into the FC2 flow cell at a flow rate of 5 µL min^−1^, yielding an immobilization level of approximately 3985 RU. Residual activated groups were blocked with ethanolamine, and FC1 was used as the reference channel. Cyclic peptide samples were prepared as stock solutions in DMSO and serially diluted with running buffer to generate multiple concentration gradients for *K*_D_ determination, with a zero-concentration sample included as the blank control. Samples were injected at a flow rate of 30 µL min^−1^, with association and dissociation phases of 120 s each. After each cycle, the sensor surface was regenerated using pH 3.0 glycine-HCl.

Sensorgrams were processed by reference subtraction and blank correction, followed by global fitting to a 1 : 1 Langmuir binding model to derive the association rate constant (*k*_a_), dissociation rate constant (*k*_d_), and equilibrium dissociation constant (*K*_D_ = *k*_d_/*k*_a_).

### Molecular dynamics simulations

Molecular dynamics simulations were carried out using Amber, and system setup was performed with AmberTools.^34,35^ The protein was described using the ff19SB force field,^36^ and the complex was solvated in an OPC water box with a 10 Å buffer and neutralized by adding Na^+^ ions.^37^ The peptide ligand was modeled as a head-to-tail cyclic peptide by forming a bond between the N atom of the first residue and the C atom of the last residue in tleap. Energy minimization was performed in two stages: an initial 6000-cycle minimization (3000 steps of steepest descent followed by 3000 steps of conjugate gradient) with positional restraints of 10.0 kcal mol^−1^ Å^−2^ on the solute, followed by a 10 000-cycle unrestrained minimization (5000 steps of steepest descent and 5000 steps of conjugate gradient). The system was then heated from 0 to 300 K over 50 ps under NVT conditions using Langevin dynamics with a collision frequency of 2.0 ps^−1^ and harmonic restraints of 2.0 kcal mol^−1^ Å^−2^ on the solute. A subsequent 50 ps density-equilibration step was conducted under NPT conditions at 300 K and 1 atm with the same restraints, followed by 500 ps of unrestrained NPT equilibration. Finally, a 100 ns production simulation was performed at 300 K and 1 atm with a 2 fs time step. All bonds involving hydrogen atoms were constrained. A 10 Å cutoff was used during minimization, and an 8 Å cutoff was used during heating, equilibration, and production.

### Trajectory analysis and MM/GBSA calculations

Production trajectories were processed using CPPTRAJ after autoimaging.^38^ Heavy-atom RMSDs of the receptor, ligand, and the entire complex were calculated relative to the first saved frame of the production trajectory. Binding free energies were estimated using the MM/GBSA workflow implemented in AmberTools.^39^ Solvent molecules and ions were removed to generate separate complex, receptor, and ligand topologies for end-state calculations. The generalized Born model was applied with igb = 5, and a salt concentration of 0.150 M.^40^ Entropic contributions were not included in the present calculations.

## Results and discussion

### Performance of HFGuidedDesign on multiple protein targets

To systematically evaluate the capability of HFGuidedDesign in designing cyclic peptide binders across diverse protein targets, we select cyclic peptide–protein complexes from the HighFold test set and the target set evaluated in RFpeptides, retaining only those with cyclic peptide ligands of no more than 20 amino acids as design targets. The selected targets include enzymes with deep and well-defined active-site clefts, such as Trypsin,^41^ Matriptase,^42^ KLK4,^43^ and Plasmin;^44^ non-enzymatic proteins featuring deep pocket-like protein–protein interaction interfaces, including MDM2 (ref. 45) and MCL-1;^46^ as well as proteins with shallow or extended binding surfaces, including KEAP1,^47^ KRAS,^48^ SPF45,^49^ AMA1,^50^ GABARAP,^51^ and Sortase A.^52^ The corresponding peptide ligands vary in length from 6 to 16 amino acids, encompassing short motif-like binders as well as longer cyclic peptides engaging extended interfaces. Detailed information on the protein targets is provided in Table S1.

**Fig. 2:**
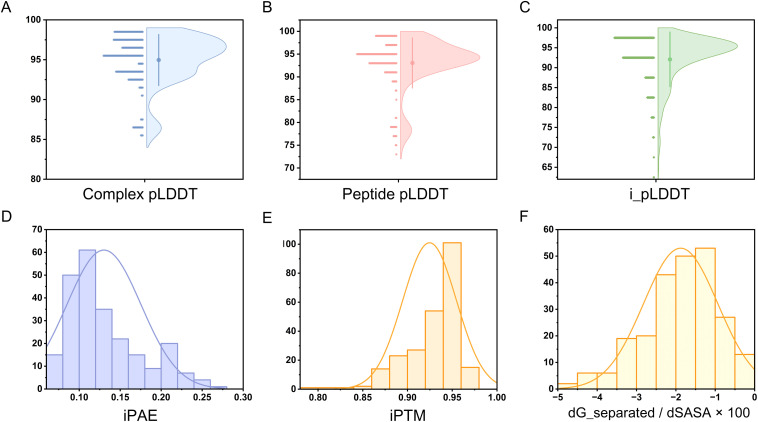
Overall distribution of structural confidence and interface quality metrics for cyclic peptide–protein complexes across 12 targets generated by HFGuidedDesign. (A) Distribution of the overall pLDDT scores of cyclic peptide–protein complexes; (B) distribution of peptide pLDDT scores for the cyclic peptide ligands; (C) distribution of interface pLDDT (i_pLDDT) scores at the peptide–protein interfaces; (D) distribution of the interface predicted aligned error (iPAE); (E) distribution of the interface predicted TM-score (iPTM), reflecting the confidence in the predicted interface geometry; (F) distribution of the interface energy metric dG_separated/dSASA × 100, calculated using the Rosetta Interface Analyzer. All metrics, including pLDDT, peptide pLDDT, i_pLDDT, iPAE, and iPTM, were obtained from HighFold.

**Fig. 3:**
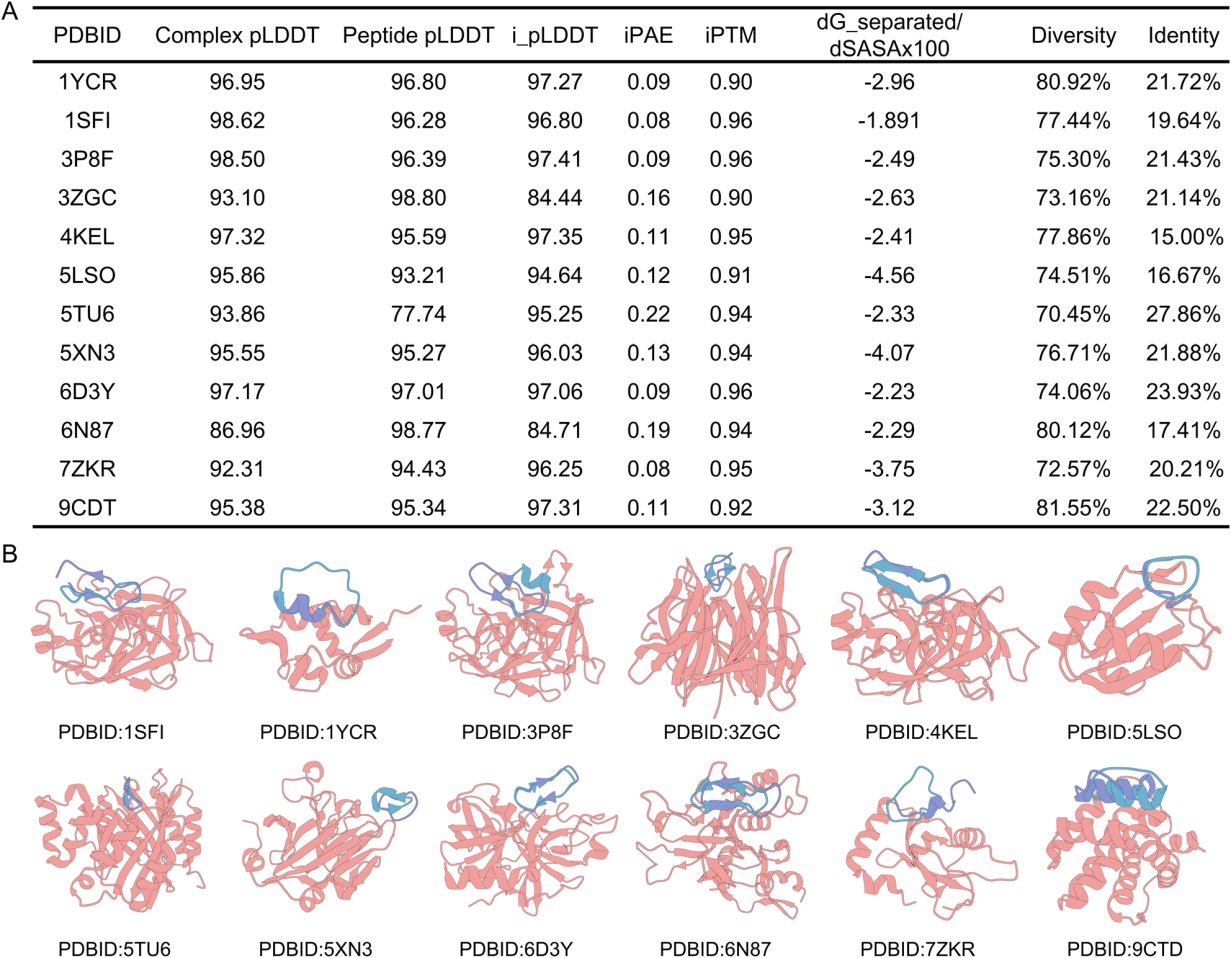
Cyclic peptide design results of HFGuidedDesign across 12 targets. (A) Performance of the top-1 designed sequence for each target across different evaluation metrics, together with the overall diversity among all generated sequences and the identity between the generated sequences and the corresponding native ligand sequences of the target proteins; (B) structural similarity between the top-1 designed cyclic peptide and the native ligand in the corresponding reference complex. The designed cyclic peptide is shown in blue, whereas the native ligand is shown in purple. The evaluation metrics were obtained from HighFold and the 3D complex structures shown in panel B were also predicted by HighFold.

For each target, HFGuidedDesign was used to generate an equal number of cyclic peptide sequences, ensuring consistency in cross-target comparisons. The distributions of the generated cyclic peptides with respect to structural and energy-related metrics are summarized in Fig. 2. Experimental results demonstrate that HFGuidedDesign consistently generates high-quality cyclic peptide sequences across all 12 targets. Structural predictions of the generated peptide–protein complexes indicate that the generated sequences can fold into binding conformations with high structural confidence and stable interfaces. The predicted complexes exhibit an average overall pLDDT of 94.9, with a mean peptide pLDDT of 93.1. The average iPTM reaches 0.92, indicating a high confidence in the predicted interface geometry, while the mean iPAE remains low at 0.13. In addition, the interface energy metric dG_separated/dSASA × 100, computed using the Rosetta Interface Analyzer, yields an average value of −1.88, further suggesting favorable interfacial interactions. Collectively, these metrics indicate that HFGuidedDesign not only produces structurally reliable cyclic peptide sequences but also enables the formation of stable, well-optimized peptide–protein interfaces. As illustrated in Fig. 3B, using the KLK4–SFTI complex (PDB ID: 4KEL) as an example, incorporation of the structure-guiding module enables HFGuidedDesign to actively generate cyclic peptide sequences containing the key pocket-recognition motif “VNQR”, which corresponds to the most critical binding fragment for target engagement.^53^ Notably, the predicted binding conformation of the designed cyclic peptide closely overlaps with that of the experimentally characterized binder, indicating that HFGuidedDesign can accurately recover key sequence-level determinants together with their corresponding three-dimensional binding modes.

In addition, sequence-level analyses were conducted on the generated cyclic peptide binders. Since cyclic peptides lack a fixed start residue, sequence similarity between two cyclic peptides is computed using a circular alignment strategy. For a pair of cyclic peptide sequences, all possible cyclic shifts of both sequences were enumerated and independently aligned against each other. For identity, the maximum sequence identity across all cyclic shifts was retained. For diversity, which is based on Levenshtein distance, the minimum edit distance across all cyclic shifts was computed and normalized by the sequence length to obtain the final diversity score. Using this strategy, the results show that HFGuidedDesign generates cyclic peptides that exhibit a high degree of diversity across the sequence space. The average diversity, quantified using the Levenshtein distance, reaches 76.22%, while the average identity to the corresponding native ligands is approximately 20.78% (Fig. 3A). This indicates that the model can generate cyclic peptide binders with highly overlapping binding conformations while maintaining high sequence novelty.

### Performance comparison of HFGuidedDesign with RFpeptides and AfCycDesign in cyclic peptide design

To further assess the performance of HFGuidedDesign in cyclic peptide design, we conducted a comparative evaluation against two representative baseline methods, RFpeptides and AfCycDesign. RFpeptides is a structure-based denoising diffusion approach that generates cyclic peptide backbone conformations complementary to the target protein surface, followed by sequence design using ProteinMPNN. In contrast, AfCycDesign employs a backpropagation-based optimization strategy built upon AlphaFold2 to directly generate cyclic peptide sequences complementary to the target proteins, with subsequent refinement by ProteinMPNN to improve sequence quality and conformational stability.

Both baseline models were systematically evaluated on the same set of 12 targets. For RFpeptide, we generate 200 cyclic peptide backbones per target, followed by sequence design using ProteinMPNN to produce 5 sequences per backbone, yielding approximately 12 000 cyclic peptide sequences in total. For AfCycDesign, we generated 1000 cyclic peptide sequences per target. In contrast, HFGuidedDesign directly generates 20 cyclic peptide sequences per target, and all generated sequences were retained for evaluation. Finally, for each target, the top 20 cyclic peptide sequences ranked by a composite structural score were selected, and their average metrics were calculated for cross-method comparison.

**Fig. 4:**
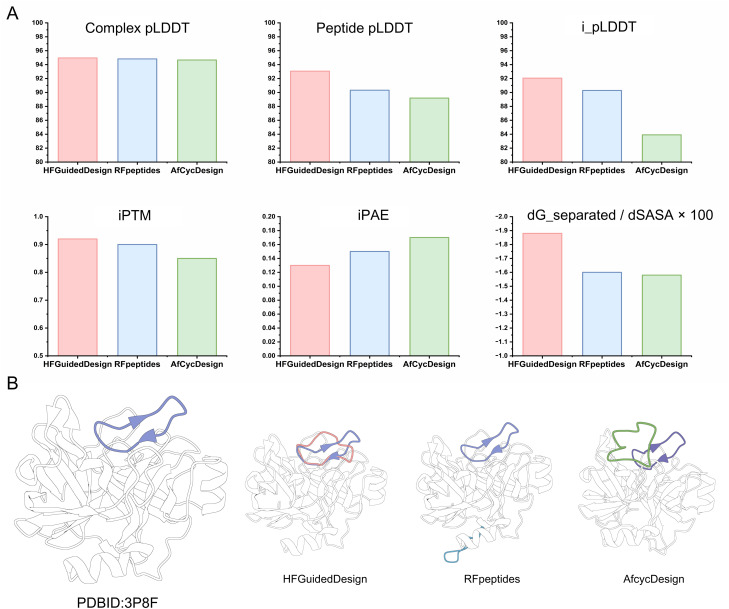
Performance comparison of HFGuidedDesign with Rfpeptides and AfCycDesigns on cyclic peptide design tasks. (A) Average values of complex pLDDT, peptide pLDDT, i_pLDDT, iPTM, iPAE, and Δ*G*_separated/dSASA × 100 for cyclic peptides designed by each model across 12 targets. (B) Representative designs for PDB ID: 3P8F. The cyclic peptide generated by HFGuidedDesign is stably positioned at the target interface, whereas peptides generated by RFpeptides and AfCycDesign show pronounced deviations from the expected binding pocket. All metrics shown in panel (A) were obtained from HighFold predictions, and the 3D complex structures shown in panel (B) were also predicted by HighFold.

As shown in Fig. 4A, the results demonstrate that HFGuidedDesign outperforms both baseline methods across all key evaluation metrics. In terms of overall complex structural quality, the cyclic peptide–protein complexes generated by HFGuidedDesign achieve an average complex pLDDT of 94.96, which is slightly higher than those obtained by RFpeptides (94.82) and AfCycDesign (94.66). Focusing on the structural quality of the cyclic peptides themselves, HFGuidedDesign yields an average peptide pLDDT of 93.06, markedly outperforming both baseline models (RFpeptides: 90.32; AfCycDesign: 89.19), indicating a stronger ability to generate structurally well-folded cyclic peptide sequences. At the interface level, HFGuidedDesign attains an average interface pLDDT(i_pLDDT) of 92.06, an average interface predicted TM-score (iPTM) of 0.92, and a low average interface predicted aligned error (iPAE) of 0.13, indicating high confidence in the predicted interface geometry. Moreover, the dG_separated/dSASA ×100, calculated using the Rosetta Interface Analyzer, reaches an average value of −1.88, further confirming that, compared with the baseline methods, cyclic peptides generated by HFGuidedDesign form peptide–protein interfaces with more favorable binding stability. Fig. 4B shows the design results of the three models for PDB ID: 3P8F. When no specific target protein hotspot residues were designated, RFpeptides and AfcycDesign generated partial cyclic peptides that deviated from the intended binding pockets, whereas HFGuidedDesign consistently positioned itself stably within the target interface region. To further assess whether the observed performance advantage was dependent on the structure predictor used for evaluation, we additionally evaluated the designed cyclic peptides using the independent structure prediction model Boltz-2. As summarized in Table 1, HFGuidedDesign consistently achieved the highest complex pLDDT, peptide pLDDT, i_pLDDT, and iPTM values, as well as the lowest iPAE values, across both HighFold and Boltz-2 evaluations. Although the absolute values differed between predictors, the relative ranking of the three methods remained largely unchanged. These results indicate that the advantage of HFGuidedDesign is not specific to the HighFold evaluation and generalizes to an independent structure predictor.

**Table 1:** Comparison of cyclic peptide design performance evaluated by HighFold and Boltz-2 across 12 targets

Metric	Predictor	HFGuidedDesign	RFpeptides	AfCycDesign
Complex pLDDT	HighFold	94.96	94.82	94.66
	Boltz-2	95.93	95.54	95.28
Peptide pLDDT	HighFold	93.06	90.32	89.19
	Boltz-2	79.86	78.60	74.74
i_pLDDT	HighFold	92.06	90.29	83.91
	Boltz-2	92.96	92.90	91.76
IPTM	HighFold	0.92	0.90	0.85
	Boltz-2	0.94	0.93	0.93
IPAE	HighFold	0.13	0.15	0.17
	Boltz-2	1.67	1.74	1.77

### *De novo* design of macrocyclic binders to MDM2 and GABARAP

To further validate the practical applicability of HFGuidedDesign, we apply HFGuidedDesign to two biologically relevant target proteins, MDM2 and GABARAP, for the *de novo* design of cyclic peptide binders.

We first focus on MDM2 as a representative target. MDM2, as an E3 ubiquitin ligase, is a key regulator of p53 stability and activity, forming a tight negative feedback regulatory loop.^54^ Overexpression of MDM2 significantly suppresses p53-induced cell cycle arrest and apoptosis.^55^ Clinically, amplification of the MDM2 gene leads to protein overexpression in approximately 30% of human osteosarcomas and soft tissue sarcomas. In tumors lacking MDM2 amplification, aberrant activation of MDM2 caused by silencing of ARF similarly results in functional inactivation of p53. Thus, MDM2 serves as a core mechanism for p53 functional tolerance in approximately 50% of tumors harboring wild-type p53, making it an attractive target for developing novel anticancer therapeutics.^56,57^ We use HFGuidedDesign to generate 20 cyclic peptide sequences targeting MDM2.

**Fig. 5:**
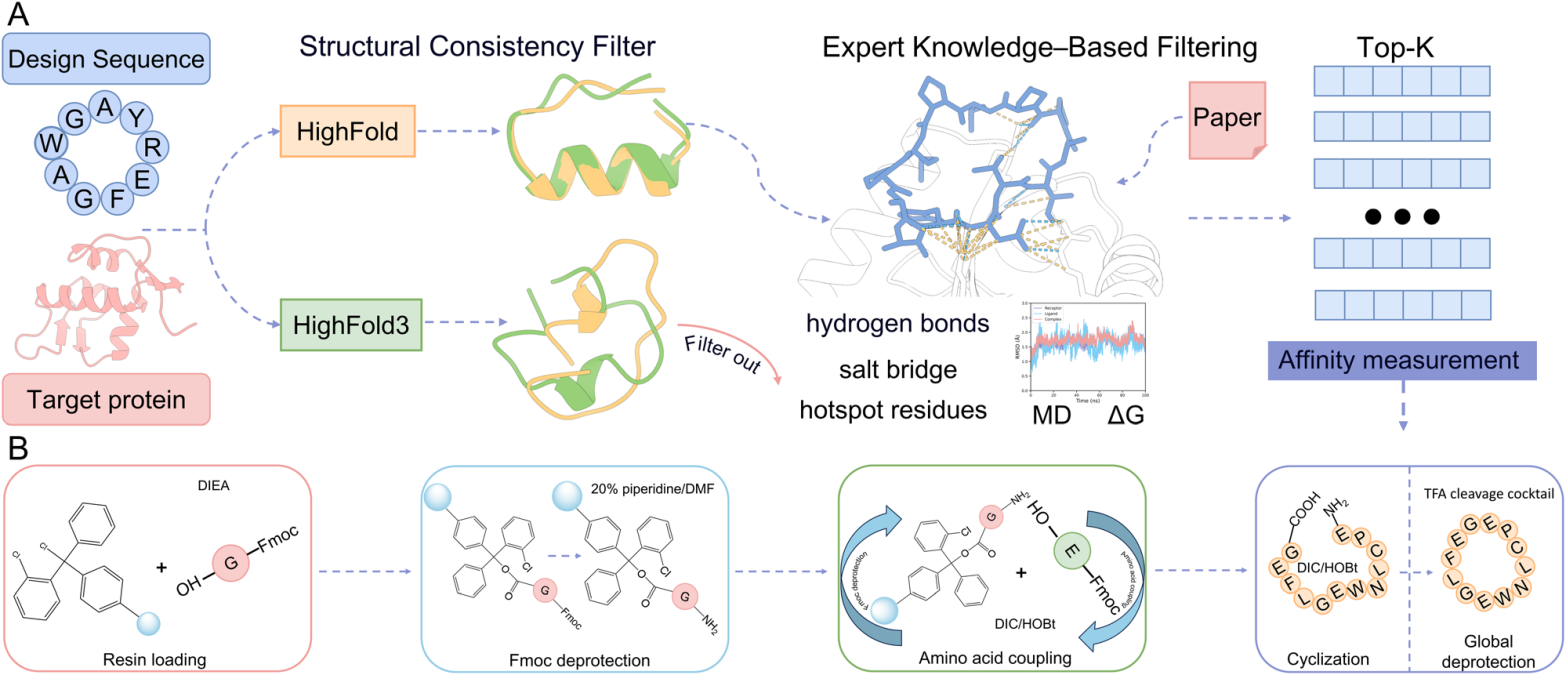
Experimental screening and synthesis of cyclic peptides designed by HFGuidedDesign. (A) Overview of the screening pipeline used to select HFGuidedDesign-generated peptides for experimental validation. The workflow integrates structural consistency assessment across multiple structure prediction models, molecular dynamics (MD) simulations, and expert knowledge derived from the literature to identify candidates for downstream experiments. (B) Schematic illustration of the chemical synthesis of the selected cyclic peptides using Fmoc-based solid-phase peptide synthesis.

**Fig. 6:**
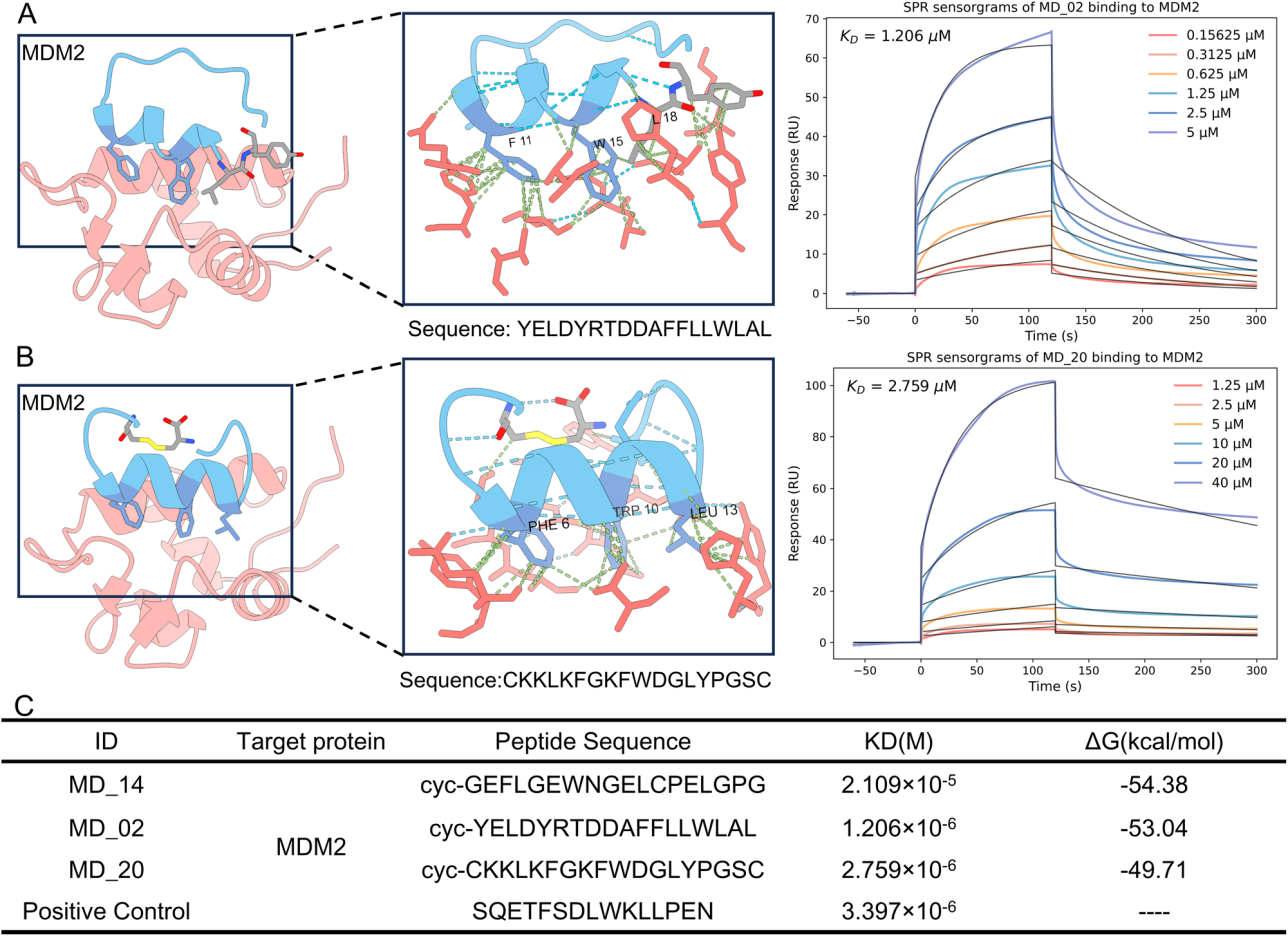
Structural and SPR characterization of generated cyclic peptides targeting MDM2. (A) Structural analysis of MD_02 in complex with MDM2 and the corresponding multi-concentration SPR sensorgrams. (B) Structural analysis of MD_20 in complex with MDM2 and the corresponding multi-concentration SPR sensorgrams. (C) Summary of the SPR-derived binding affinities of the generated peptides and the positive control derived from the p53 transactivation domain, together with MD trajectory-based end-point binding free energy estimates for the generated peptides.

To prioritize candidates for experimental validation, we subject these sequences to a multi-step *in silico* screening and ranking pipeline. As shown in Fig. 5A, we first perform complex structure prediction for each designed cyclic peptide in complex with the target protein using both HighFold and HighFold3,^58^ and assess the consistency of the predicted peptide conformations between the two models. We retain only those cyclic peptides for which HighFold and HighFold3 predict highly concordant complex structures, thereby increasing confidence in their structural plausibility. Next, we conduct a detailed interaction analysis between the filtered cyclic peptides and MDM2 using standard molecular visualization and protein–protein interaction analysis tools,^59^ informed by prior structural and biochemical knowledge reported in the literature. This analysis focuses on key interfacial interactions, including hydrogen bonding networks, hydrophobic contacts, and shape complementarity. In parallel, we perform energetic and stability assessments using Rosetta Interface Analyzer as well as molecular dynamics (MD) simulations to further evaluate interface quality and conformational robustness. Based on an integrated ranking that jointly considers structural consistency, interfacial interactions, energetic favorability, dynamic stability, and synthetic accessibility, the top three cyclic peptide sequences were selected for experimental validation. The selected peptides are synthesized using standard Fmoc solid-phase peptide synthesis (Fmoc-SPPS) and evaluated for binding affinity using SPR. Among the three head-to-tail amide-cyclized peptides, one fails during chemical synthesis, while the remaining two exhibit micromolar binding affinity toward MDM2. The sequences of the MDM2-targeting cyclic peptides are listed in Table S3, and their analytical HPLC and mass spectrometric characterization data are provided in Fig. S3–5. The best-performing design demonstrated a binding affinity of 1.206 µM in SPR measurements, as shown in Fig. 6A. To further validate the applicability of HFGuidedDesign in *de novo* design of head-to-tail cyclic peptides mediated by a disulfide bond, we design and synthesize an additional peptide of this class for affinity assessment. As shown in Fig. 6B, this disulfide-bridged cyclic peptide exhibited a dissociation constant (*K*_D_) of 2.759 µM, supporting the capability of our approach across different cyclization chemistries. We further analyze the sequence and structural features of the most active cyclic peptide against MDM2, YELDYRTDDAFFLLWLAL, as shown in Fig. 6. This peptide forms a stable cyclic backbone through head-to-tail amide cyclization. Notably, the cyclic peptide bears a hydrophobic-rich segment DAFFLLWLAL within its backbone, which comprises multiple aromatic and aliphatic residues, including phenylalanine (Phe), tryptophan (Trp), leucine (Leu), and alanine (Ala). These residues provide a favorable interaction module for recognizing the deep hydrophobic cleft of MDM2. In the predicted complex structure, the hydrophobic residues are oriented toward the MDM2 binding pocket and establish extensive hydrophobic contacts with pocket-lining residues. This binding pattern is consistent with the canonical p53-MDM2 recognition mode, in which the key anchoring residues of the p53 transactivation domain, Phe19, Trp23, and Leu26, insert into the hydrophobic pocket of MDM2. Compared with the native p53 transactivation helix, the designed cyclic peptide adopts a more compact and conformationally constrained topology. This feature is mainly attributed to the conformational restriction imposed by backbone cyclization, together with the spatial distribution of polar and charged residues, such as tyrosine (Tyr), glutamate (Glu), aspartate (Asp), and arginine (Arg), around the cyclic scaffold. These residues help stabilize the intramolecular conformation and enhance interfacial complementarity with the target protein. The constrained topology also reduces the conformational entropy penalty upon binding, enabling the peptide to engage the MDM2 pocket with favorable binding geometry. Molecular dynamics (MD) simulations further support the structural stability and binding energetics of the designed peptide-MDM2 complex. Throughout a 100 ns simulation, the backbone RMSD of the peptide, the receptor, and the overall complex remain stable, with no evidence of large-scale conformational drift, indicating a persistent and well-defined binding mode. In addition, end-point free energy analysis based on the MD trajectories yields a favorable binding free energy of Δ*G* = −53.04 kcal mol^−1^, further supporting the strong and stable interaction between the designed cyclic peptide and MDM2. The detailed molecular dynamics simulation results for the MDM2 complex are provided in Section 6 of the SI.

Following successful validation of HFGuidedDesign's capability to design cyclic peptide sequences for the MDM2 target, we further applied it to the GABARAP protein. Unlike the MDM2 binding pocket, which is composed entirely of α-helices, the GABARAP binding site features a combination of α-helices and β-sheets, exhibiting significantly different structural characteristics.GABARAP (γ-aminobutyric acid type A receptor-associated protein) is a member of the ATG8 protein family and plays a central role in autophagy by mediating autophagosome maturation and cargo recruitment. GABARAP recognizes short linear interaction motifs, commonly referred to as LC3-interacting region (LIR) motifs, in a wide range of binding partners and engages them through a conserved hydrophobic groove on its surface. These interactions are essential for selective autophagy and intracellular trafficking processes.^60–62^

**Fig. 7:**
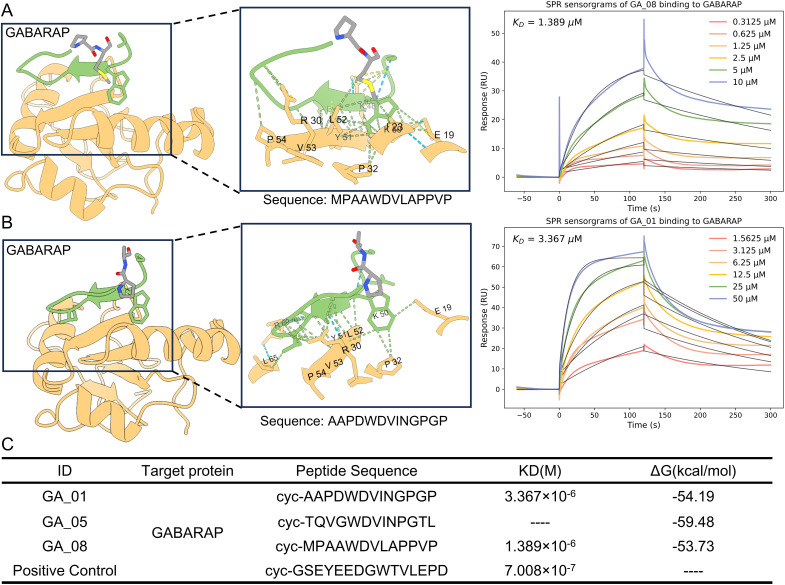
Structural and SPR characterization of generated cyclic peptides targeting GABARAP. (A) Structural analysis of GA_08 in complex with GABARAP and the corresponding multi-concentration SPR sensorgrams. (B) Structural analysis of GA_01 in complex with GABARAP and the corresponding multi-concentration SPR sensorgrams. (C) Summary of the SPR-derived binding affinities of the generated peptides and the positive control derived from RFpeptides, together with MD trajectory-based end-point binding free energy estimates for the generated peptides.

For the GABARAP target, the same computational design and screening pipeline established for MDM2 was applied. First, 20 cyclic peptide candidates were generated using HFGuidedDesign, followed by multi-criteria ranking based on structural prediction consistency, binding interface interactions, and molecular dynamics simulation stability. The top three ranked sequences were selected for synthesis *via* standard Fmoc-SPPS and subsequently evaluated for binding affinity by SPR. The sequences of the GABARAP-targeting cyclic peptides are listed in Table S3, and their analytical HPLC and mass spectrometric characterization data are provided in Fig. S8–10. As shown in Fig. 7, two of the three designed cyclic peptides demonstrated reliable concentration-dependent binding to GABARAP, corresponding to a 66.7% experimental success rate.

We also analyze the sequence and structural features of the most active cyclic peptide binder targeting GABARAP, MPAAWDVLAPPVP. The sequence contains a canonical LIR-like motif, W–D–V–L, which closely matches the consensus [W/F/Y]–X–X–[L/I/V] pattern recognized by members of the ATG8 protein family, including GABARAP. In the predicted complex structure, the aromatic residue Trp is positioned to insert into the conserved W-site hydrophobic pocket of GABARAP, while Leu occupies the adjacent L-site pocket, forming key hydrophobic contacts that stabilize the interaction. Meanwhile, the Asp residue within the motif further stabilizes the binding interface through polar interactions with neighboring residues on the protein surface. End-point binding free energy analysis based on molecular dynamics trajectories also yields a favorable binding free energy of Δ*G* = −53.73 kcal mol^−1^, further supporting the strong and stable interaction between the designed cyclic peptide and GABARAP. The detailed molecular dynamics simulation results for the GABARAP complex are provided in Section 9 of the SI.

### Validation on unseen targets and sequence novelty analysis

To evaluate the ability of HFGuidedDesign to generate cyclic peptide binders for targets not present in the peptide–protein complex dataset used for discrete diffusion model training, we apply the method to CD28 (ref. 63) and SPIRE1.^64^ For both targets, we employed the same computational design and screening pipeline previously established for MDM2 and GABARAP. Specifically, HFGuidedDesign generated 20 cyclic peptide candidates per target, which were then subjected to multi-criteria ranking based on structural prediction consistency, binding interface interactions, and Rosetta Interface Analyzer scores. The top three ranked sequences for each target were selected for MM/GBSA binding free energy calculations. As summarized in Table 2, all selected cyclic peptides exhibited favorable predicted binding affinities toward both targets. The binding free energies estimated from MD simulations ranged from −48.82 to −54.47 kcal mol^−1^ for CD28-targeting peptides and from −37.25 to −58.49 kcal mol^−1^ for SPIRE1-targeting peptides. These values suggest that HFGuidedDesign can generate cyclic peptides with promising binding potential even for targets absent from the training dataset. To further verify that HFGuidedDesign does not simply memorize and reproduce sequences from the peptide–protein complex dataset, we also analyze the sequence identity between the cyclic peptides produced by HFGuidedDesign and the corresponding training ligand sequences in PPIKB across all 12 benchmark targets. As shown in Table 3, the sequence identity ranged from 21.60% to 46.44%, with an overall mean of approximately 31.00%. These relatively low identity values indicate that the generated peptides are substantially distinct from the training ligands. Collectively, these results demonstrate that HFGuidedDesign can generate novel cyclic peptide candidates with favorable predicted binding affinities for unseen targets, without simply recapitulating known sequences from the training set.

**Table 2:** Computational validation of HFGuidedDesign-designed cyclic peptides targeting CD28 and SPIRE1

Target protein	Peptide sequence	Δ*G* (kcal mol^−1^)
CD28	cyc-AGMSQVAALVWEEF	−54.47
	cyc-VLLPESWALVRPED	−50.20
	cyc-ALLSERAALVDEEL	−48.82
SPIRE1	cyc-PEARLRERVAAKEG	−39.49
	cyc-IRERIETLQPELDR	−37.25
	cyc-EAAATQKRLAHKYLGE	−58.49

**Table 3:** Sequence identity between HFGuidedDesign-generated cyclic peptide sequences and training ligand sequences associated with the test targets collected from the dataset

PDBID	Protein name	Design length	Average identity
1SFI	Trypsin	14	21.60%
1YCR	MDM2	15	36.96%
3P8F	Matriptase	14	29.02%
3ZGC	KEAP1	7	46.44%
4KEL	KLK4	14	23.57%
5LSO	SPF45	6	38.69%
5TU6	PagF	7	23.71%
5XN3	SPSB2	8	29.96%
6D3Y	Plasmin	14	25.00%
6N87	AMA1	13	31.52%
7ZKR	GABARAP	13	32.74%
9CDT	MCL-1	16	29.83%

## Conclusions

In this study, we present HFGuidedDesign, a deep learning framework for *de novo* design of cyclic peptide binders given only the target protein sequence as input. HFGuidedDesign integrates a discrete diffusion model operating in protein sequence space with an external structure-guiding mechanism, in which HighFold is introduced as a real-time structural evaluation module during the reverse diffusion process. By dynamically steering the generative trajectory toward ensembles of cyclic peptide sequences with enhanced structural plausibility and binding potential, HFGuidedDesign effectively addresses several longstanding challenges in cyclic peptide design, including the scarcity of cyclic peptide–protein complex data, the difficulty of maintaining structural plausibility during sequence generation, and the low success rates of conventional two-stage design pipelines.

Systematic evaluation across 12 diverse protein targets demonstrates that HFGuidedDesign consistently generates cyclic peptide binders with high structural confidence, well-defined binding interfaces, and favorable binding energetics. On average, the resulting peptide–protein complexes achieve a complex pLDDT of 94.9, a peptide pLDDT of 93.1, an iPTM of 0.92, an iPAE of 0.13, and an interface energy metric (dG_separated/dSASA × 100) of −1.88. Compared with representative baseline methods, including RFpeptides and AfCycDesign, HFGuidedDesign exhibits substantial improvements across key metrics of structural quality, interface stability, and peptide folding confidence. Simultaneously, the generated cyclic peptides remain highly novel, sharing only 20.78% average sequence identity with native ligands, while maintaining strong sequence diversity, as reflected by an average Levenshtein distance of 76.22%.

Beyond *in silico* validation using computational metrics, we further applied HFGuidedDesign to two biologically and therapeutically relevant proteins: MDM2, a master negative regulator of the p53 tumor suppressor signaling pathway, and GABARAP, a core autophagy-related protein involved in cancer progression and cellular homeostasis. Cyclic peptides designed for both targets were synthesized using standard solid-phase peptide synthesis protocols, and their binding affinities were evaluated by surface plasmon resonance (SPR). The experimental results showed that 75% and 66.7% of the designed peptides exhibited detectable binding activity toward MDM2 and GABARAP, respectively. Among these candidates, the best-performing peptide displayed a binding affinity of 1.206 µM for MDM2, while a peptide targeting GABARAP achieved a binding affinity of 1.389 µM. These results demonstrate the design capability of HFGuidedDesign, indicating that this structure-guided discrete diffusion framework not only generates computationally plausible sequences but also efficiently produces cyclic peptide sequences with experimentally validated binding activity.

HFGuidedDesign also establishes a flexible and extensible template for cyclic peptide design. The modular nature of the guidance mechanism enables the incorporation of additional objective functions, such as solubility, membrane permeability, and metabolic stability scores, thereby allowing multi-property optimization within a unified generative framework. Beyond the current focus on canonical cyclic peptides, future versions of this framework could be further extended to chemically more diverse cyclic peptide scaffolds. By incorporating broader residue and linker types and coupling the generation process with structure prediction modules capable of handling user-defined covalent connectivity, the model could support more complex cyclization patterns, such as organic linkers and noncanonical cyclization bonds.

Despite the achievements outlined above, several limitations warrant attention. First, the current implementation relies heavily on the accuracy of the external structure predictor HighFold. While HighFold demonstrates high sensitivity toward key binding residues and conformational changes, its performance is inherently constrained by the quality and diversity of available cyclopeptide–protein complex data. As structural databases expand and prediction algorithms improve, the reliability of the steering module is expected to increase accordingly. Second, the current framework does not explicitly accept user-defined binding-site information, such as hotspot residues or predefined pocket residues, as input, and therefore does not directly enforce peptide binding to a predefined functional site. Instead, binding-site preference is introduced implicitly through HighFold-based structure-guided generation and interface-confidence metrics such as iPTM and iPAE. Although this strategy frequently guides cyclic peptides toward biologically relevant interaction regions, it cannot guarantee precise targeting of a user-defined orthosteric or allosteric site, particularly for proteins containing multiple potential binding surfaces. Future work will aim to incorporate explicit site-conditioning information into the generation process. For example, user-specified hotspot or pocket residues could be encoded as additional constraints, and peptide-site distance- or contact-based terms could be integrated into the guidance objective to improve controllability in site-specific cyclic peptide design. Third, the computational cost of performing real-time structural evaluation during diffusion remains significant, particularly for large-scale design tasks or longer peptide segments. Future work could explore more efficient steering scheduling strategies, approximate scoring functions, or knowledge distillation approaches to accelerate the sampling process without compromising design quality.

In summary, HFGuidedDesign advances *de novo* cyclic peptide design by integrating sequence-space discrete diffusion with real-time structural feedback, offering a scalable platform to facilitate the discovery of cyclic peptide therapeutics for challenging and intractable targets.

## Author contributions

Haomeng Hu: conceptualization, writing – original draft, methodology, data curation, investigation, visualization, software, formal analysis. Renjie Zhu: data curation. Ning Zhu: data curation, writing – review and editing. Chengyun Zhang: writing – review and editing. Tianfeng Shang: writing – review and editing. Chongyang Li: data curation. Jingjing Guo: writing – review and editing. Xudong Wang: supervision. Hongliang Duan: resources, supervision, project administration.

## Conflicts of interest

The authors declare no competing financial interests.

## Supplementary Material

SC-017-D6SC02631A-s001

## Data Availability

The code and dataset used in this study are publicly available in the GitHub repository at https://github.com/hongliangduan/HFGuidedDesign. Supplementary information (SI) is available. See DOI: https://doi.org/10.1039/d6sc02631a.
