# Supplementary material for: HFGuidedDesign: de novo design of cyclic peptide binders via structure-guided discrete diffusion
Source: Chem Sci. 2026 Jun 30. Online ahead of print. doi: 10.1039/d6sc02631a (PMC13334703; doi:10.1039/d6sc02631a)
Supplement: SC-OLF-D6SC02631A-s001 [file SC-OLF-D6SC02631A-s001.pdf]

Supplementary Information (SI) for Chemical Science.
This journal is © The Royal Society of Chemistry 2026

Supporting Information

HFGuidedDesign: De Novo Design of Cyclic Peptide Binders via Structure-Guided Discrete Diffusion

Haomeng Hu[a], Renjie Zhu[a], Ning Zhu[b], Chengyun Zhang[b], Tianfeng Shang[c], Chongyang Li[b], Jingjing Guo[b], Xudong Wang[a,*], Hongliang Duan[b,*]

[a] College of Pharmaceutical Sciences, Zhejiang University of Technology, Hangzhou, 310014, China

[b] Faculty of Applied Sciences, Macao Polytechnic University, Macao, 999078, China

[c] AI department, Shenzhen Highslab Therapeutics. Inc, Shenzhen, 518000, China

* The corresponding authors. Email: hduan@mpu.edu.mo, xdwang2019@zjut.edu.cn

Table of Contents

Section 1. Details of the 12 protein targets used for generalizability evaluation. 2
Section 2. Computational Time-Cost Comparison of HFGuidedDesign, RFpeptides, and AfCycDesign. 3
Section 3. Independent Boltz-2-Based Evaluation of HFGuidedDesign, RFpeptides, and AfCycDesign. 4
Section 4 HFGuidedDesign-Designed Cyclic Peptide Sequences Targeting MDM2. .. 5
Section 5 Chemical Synthesis and Analytical Characterization of MDM2-Targeting Cyclic Peptides 7
Section 6 Structural Stability and SPR Binding Validation of HFGuidedDesign-Designed Cyclic Peptides Targeting MDM2 10
Section 7 HFGuidedDesign-Designed Cyclic Peptide Sequences Targeting GABARAP 11
Section 8 Chemical Synthesis and Analytical Characterization of GABARAP-Targeting Cyclic Peptides 13
Section 9 Structural Stability and SPR Binding Validation of HFGuidedDesign-Designed Cyclic Peptides Targeting GABARAP 16

Section 1. Details of the 12 protein targets used for generalizability evaluation.

Table S1. Details of the 12 protein targets used for generalizability evaluation

PDB ID	Protein Name	Protein Family	Binding Site Type	Peptide Length
1SFI	Trypsin	Serine protease	Deep canyon-like active site cleft	14
1YCR	MDM2	MDM2/MDM4 family	Deep hydrophobic α-helical groove	15
3P8F	Matriptase	Serine protease	Deep active site cleft	14
3ZGC	KEAP1	Kelch β-propeller	Shallow groove/pocket on β-propeller surface	7
4KEL	KLK4	Serine protease	Deep active site cleft	14
5LSO	SPF45	U2AF homology motif (UHM) domain	Extended/shallow PPI groove for ULM motifs	6
5TU6	PagF	Cyanobactin prenyltransferase	Solvent-exposed hydrophobic pocket completed by peptide substrate binding	7
5XN3	SPSB2	SPRY/SOCS box protein family	Shallow surface groove for iNOS DINNNV motif recognition	8
6D3Y	Plasmin	Serine protease	Deep active site cleft	14
6N87	AMA1	Apical membrane antigen 1	Hydrophobic groove for host invasion PPI	13
7ZKR	GABARAP	ATG8 ubiquitin-like family	Hydrophobic grooves (HP1 and HP2 pockets) on ubiquitin-like core	13
9CDT	MCL-1	BCL-2 family	Helical groove for BH3 motif mimic	16

Section 2. Computational Time-Cost Comparison of HFGuidedDesign, RFpeptides, and AfCycDesign.

Table S2. computational time-cost comparison of HFGuidedDesign, RFpeptides, and AfCycDesign.

Method	Main workflow	Average time per target
HFGuidedDesign	Structure-guided reverse diffusion generation	~80 h
RFpeptides	Backbone generation + ProteinMPNN sequence design + filtering	~63 h
AfCycDesign	Sequence design + ProteinMPNN optimization + filterin	~84 h

The time cost varied depending on sequence length, target difficulty, and the design workflow of each method. As summarized in Table S2, HFGuidedDesign required approximately 80 hours per target to directly generate and retain 20 cyclic peptide sequences. RFpeptides, with the diffusion step parameter set to T=50, required approximately 63 hours per target from cyclic backbone generation and sequence design to the final selection of the top 20 sequences. AfCycDesign required approximately 84 hours per target from initial sequence design, ProteinMPNN-based sequence optimization, and final filtering to the selection of the top 20 sequences.

Section 3. Independent Boltz-2-Based Evaluation of HFGuidedDesign, RFpeptides, and AfCycDesign.

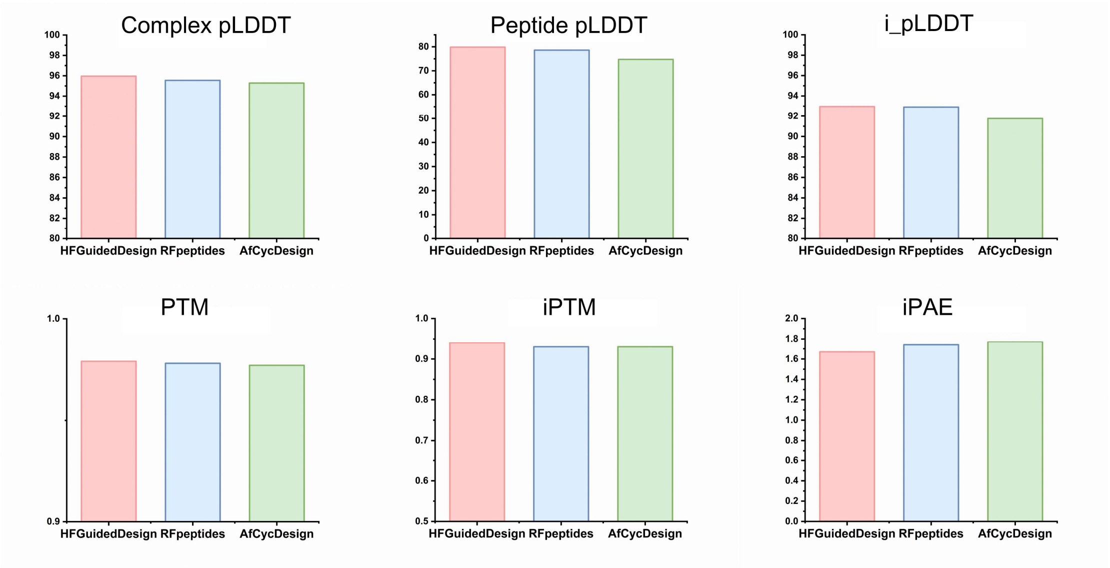

Fig.S1 Performance comparison of HFGuidedDesign with RFpeptides and AfCycDesigns on cyclic peptide design tasks. Average values of complex pLDDT, peptide pLDDT, i_pLDDT, PTM, iPTM and iPAE for cyclic peptides designed by each model across 12 targets.

Section 4 HFGuidedDesign-Designed Cyclic Peptide Sequences Targeting MDM2.

Table S3. HFGuidedDesign-Designed Cyclic Peptide Sequences Targeting MDM2.

ID	Target Protein	Length	Sequence
MD_01	MDM2	18	cyc-SWAREMSVPPPGDTQAAL
MD_02	MDM2	18	cyc-YELDYRTDDAFFLLWLAL
MD_03	MDM2	18	cyc-WETCLNEVPEWLKVEWEW
MD_04	MDM2	18	cyc-WAEASKSLAVYEEEFKSL
MD_05	MDM2	18	cyc-LLKKMLSWEWWYSKSGYN
MD_06	MDM2	18	cyc-LMYDKAAAADTFAALWWA
MD_07	MDM2	18	cyc-RTVKERGELCNCRPVDWV
MD_08	MDM2	18	cyc-HIWNSLPGALSWGASAYA
MD_09	MDM2	18	cyc-MYSAPPQSDQWRDWWLSL
MD_10	MDM2	18	cyc-YASYVDDLIDDSDTLATD
MD_11	MDM2	18	cyc-ARGALFSDYWAGLLNGRQ
MD_12	MDM2	18	cyc-EEFEACMRAVCEEAEVES
MD_13	MDM2	18	cyc-LEDRPRFEDLWCAVARE
MD_14	MDM2	18	cyc-GEFLGEWNGELCPELGPG
MD_15	MDM2	18	cyc-LSMQWVVVVDQAQVFLVL
MD_16	MDM2	18	cyc-LSNSAWHRHWNSLHRELK
MD_17	MDM2	18	cyc-AALWRSVMVGQAATSNTA
MD_18	MDM2	18	cyc-ARRRRSSDFHGSFLNSML
MD_19	MDM2	18	cyc-WKEYWQSVSTNLAPKSPR
MD_20	MDM2	18	cyc-CKKLKFGKFWDGLYPGSC

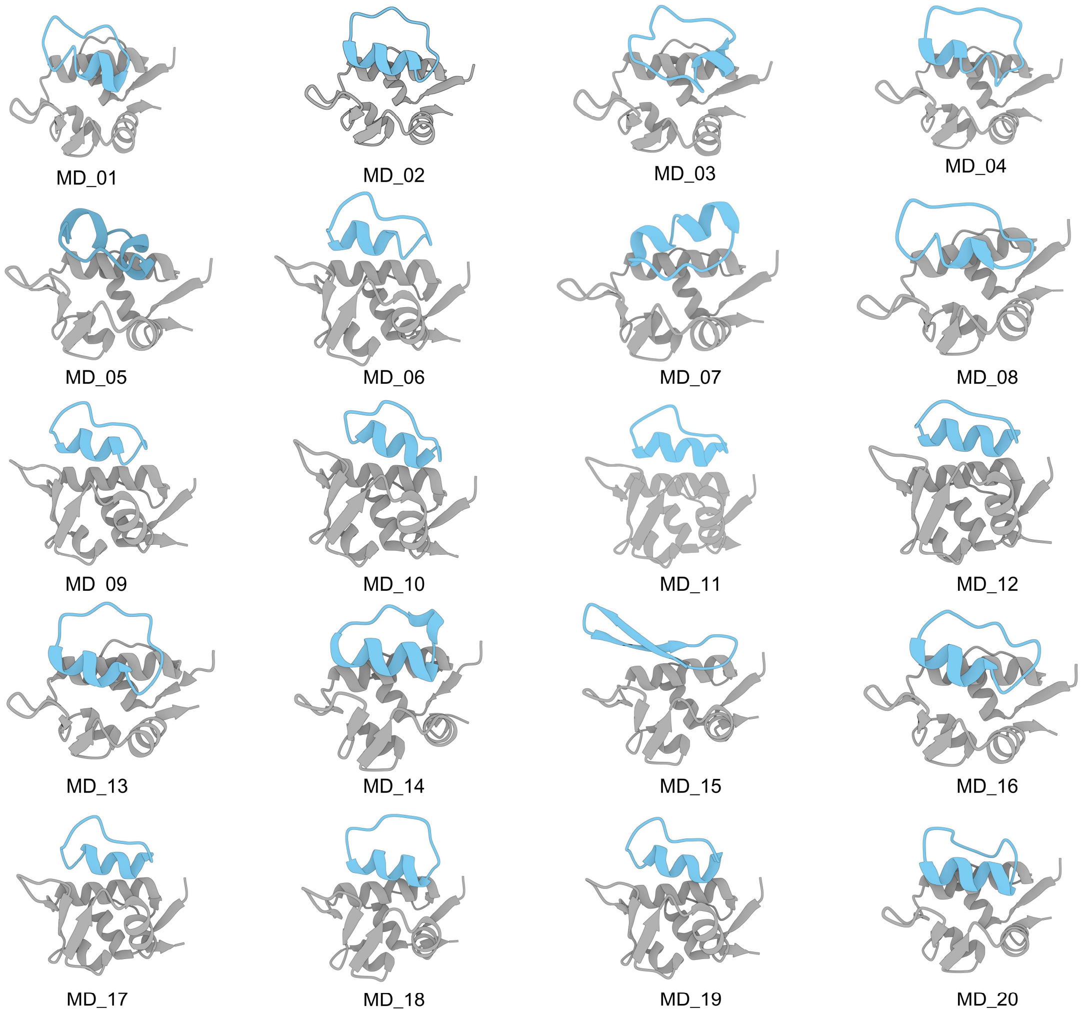

Fig.S2 Structural diversity of MDM2-binding cyclic peptides designed by HFGuidedDesign

Section 5 Chemical Synthesis and Analytical Characterization of MDM2-Targeting Cyclic Peptides

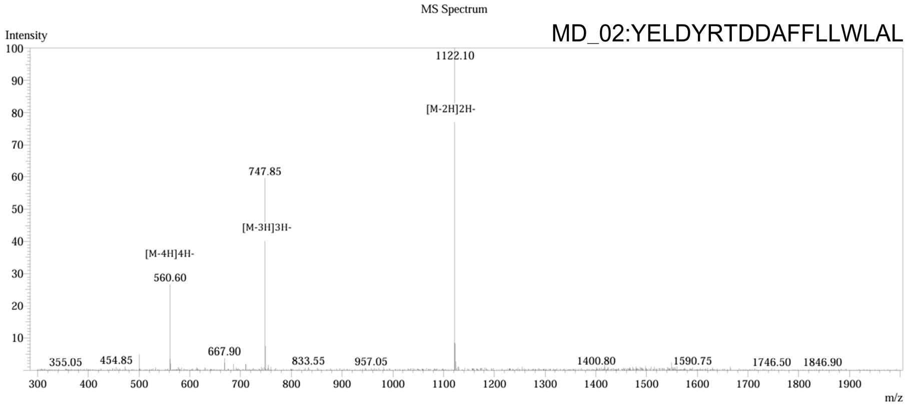

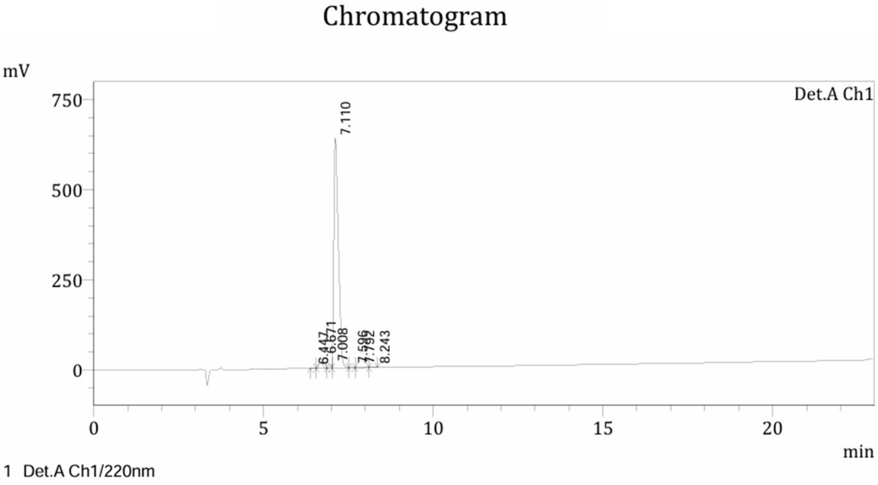

PeakTable

Detector A Ch1 220nm

Peak#	Ret. Time	Area	Height	Area %	Height %
1	6.447	3513	633	0.058	0.093
2	6.671	178942	30571	2.964	4.480
3	7.008	30317	9120	0.502	1.336
4	7.110	5785267	637773	95.819	93.457
5	7.596	13905	1951	0.230	0.286
6	7.792	20507	1437	0.340	0.211
7	8.243	5249	942	0.087	0.138
Total		6037699	682426	100.000	100.000

Fig.S3 Analytical HPLC and mass spectrometric characterization of the MDM2-targeting cyclic peptide MD_02 designed by HFGuidedDesign.

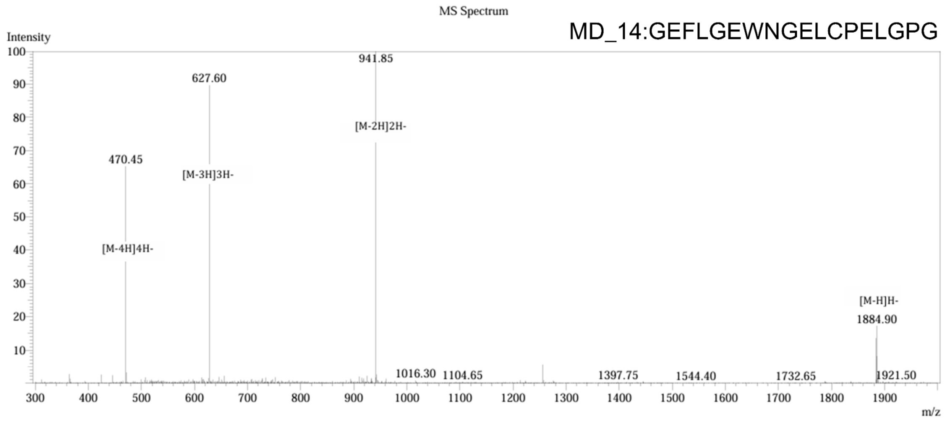

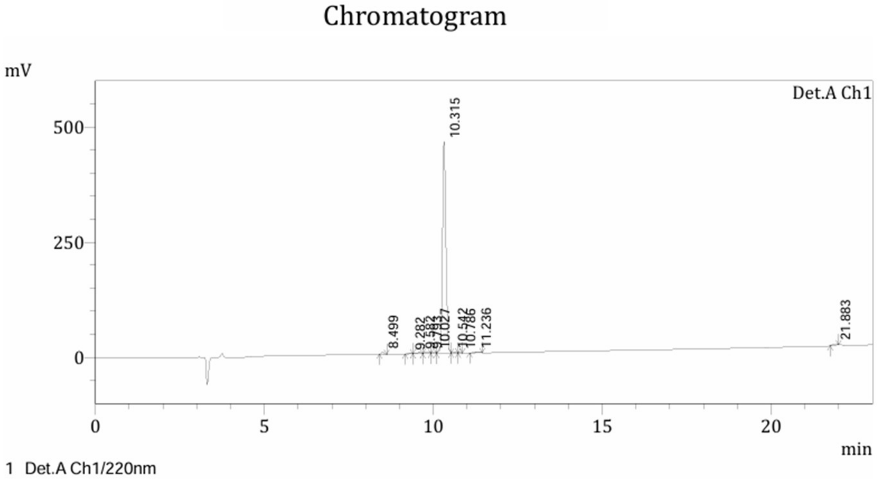

PeakTable

Detector A Ch1 220nm

Peak#	Ret. Time	Area	Height	Area %	Height %
1	8.499	24317	4244	0.731	0.885
2	9.282	4087	582	0.123	0.121
3	9.582	24580	2693	0.739	0.561
4	9.793	28703	2504	0.862	0.522
5	10.027	21929	2476	0.659	0.516
6	10.315	3163391	459118	95.045	95.699
7	10.542	16611	2320	0.499	0.484
8	10.786	2522	492	0.076	0.103
9	11.236	15086	2168	0.453	0.452
10	21.883	27097	3155	0.814	0.658
Total		3328323	479753	100.000	100.000

Fig.S4 Analytical HPLC and mass spectrometric characterization of the MDM2-targeting cyclic peptide MD_14 designed by HFGuidedDesign.

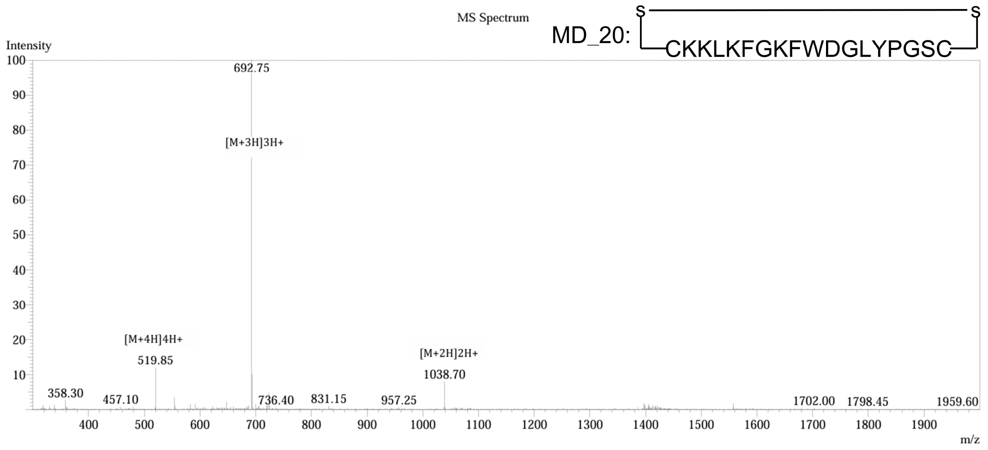

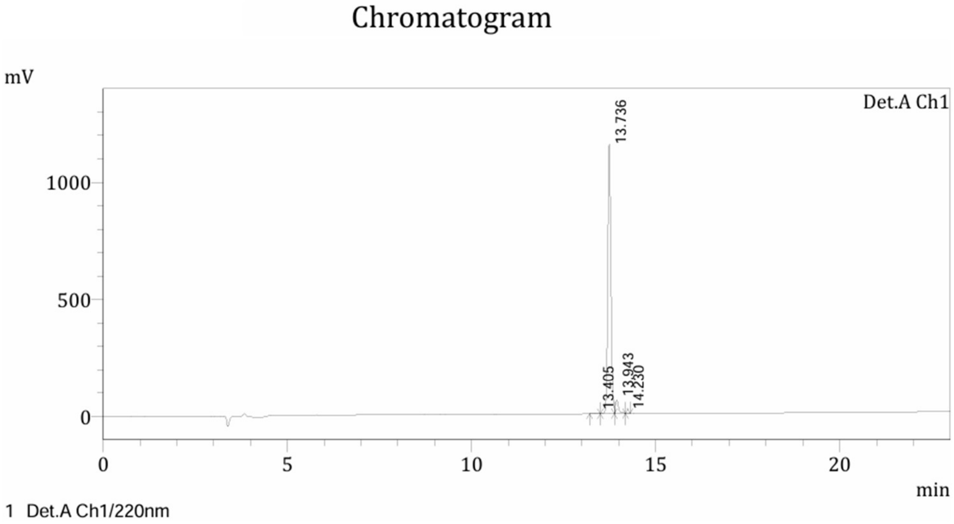

PeakTable

Detector A Ch1 220nm

Peak#	Ret. Time	Area	Height	Area %	Height %
1	13.405	8181	1108	0.113	0.091
2	13.736	6857979	1151501	95.110	95.063
3	13.943	335215	56838	4.649	4.692
4	14.230	9237	1849	0.128	0.153
Total		7210612	1211297	100.000	100.000

Fig.S5 Analytical HPLC and mass spectrometric characterization of the MDM2-targeting cyclic peptide MD_20 designed by HFGuidedDesign.

Section 6 Structural Stability and SPR Binding Validation of HFGuidedDesign-Designed Cyclic Peptides Targeting MDM2

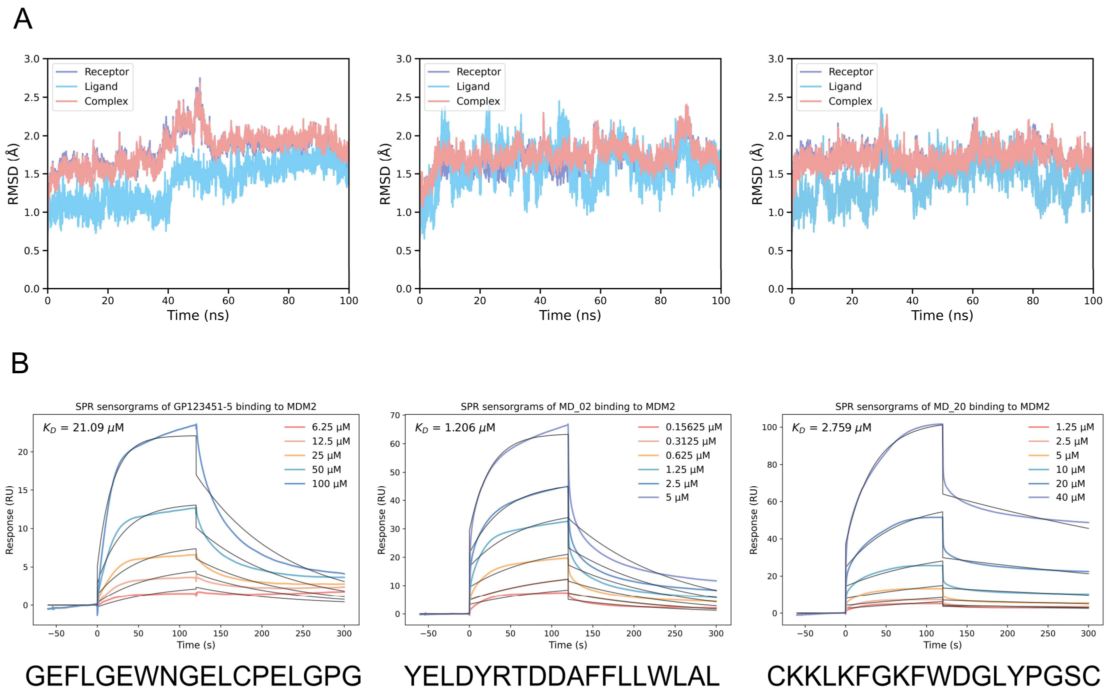

Fig.S6 Structural Stability and SPR Binding Validation of HFGuidedDesign-Designed Cyclic Peptides Targeting MDM2.

Section 7 HFGuidedDesign-Designed Cyclic Peptide Sequences Targeting GABARAP

Table S4. HFGuidedDesign-Designed Cyclic Peptide Sequences Targeting GABARAP.

ID	Target Protein	Length	Sequence
GA_01	GABARAP	13	cyc-AAPDWDVINGPGP
GA_02	GABARAP	13	cyc-GIAGWDVLYGAAT
GA_03	GABARAP	13	cyc-GMAGWNVLYAILP
GA_04	GABARAP	13	cyc-PDASWDVLTPYPC
GA_05	GABARAP	13	cyc-TQVGWDVINPGTL
GA_06	GABARAP	13	cyc-VAMCWVDWVSGRV
GA_07	GABARAP	13	cyc-YGTWWDVIKYVPA
GA_08	GABARAP	13	cyc-MPAAWDVLAPPVP
GA_09	GABARAP	15	cyc-APPSPGPTWDVLGAA
GA_10	GABARAP	15	cyc-CTWRSDANWWVLAGW
GA_11	GABARAP	15	cyc-LYNIVLDNLAIVETE
GA_12	GABARAP	15	cyc-EYIIVELNGETKEYA
GA_13	GABARAP	15	cyc-GEFVSIEEWDVLQPD
GA_14	GABARAP	15	cyc-GHWMCHPWDIWEWDP
GA_15	GABARAP	15	cyc-AGKWLKYKWDVGAWG
GA_16	GABARAP	15	cyc-PAMWAWADGWDVITM
GA_17	GABARAP	15	cyc-RMMPDWDVLRDDYAQ
GA_18	GABARAP	15	cyc-GKWNELPFTVLVWVE
GA_19	GABARAP	15	cyc-WKVKAVKDWCVVMAG
GA_20	GABARAP	15	cyc-GEWDVLDGACQVLFS

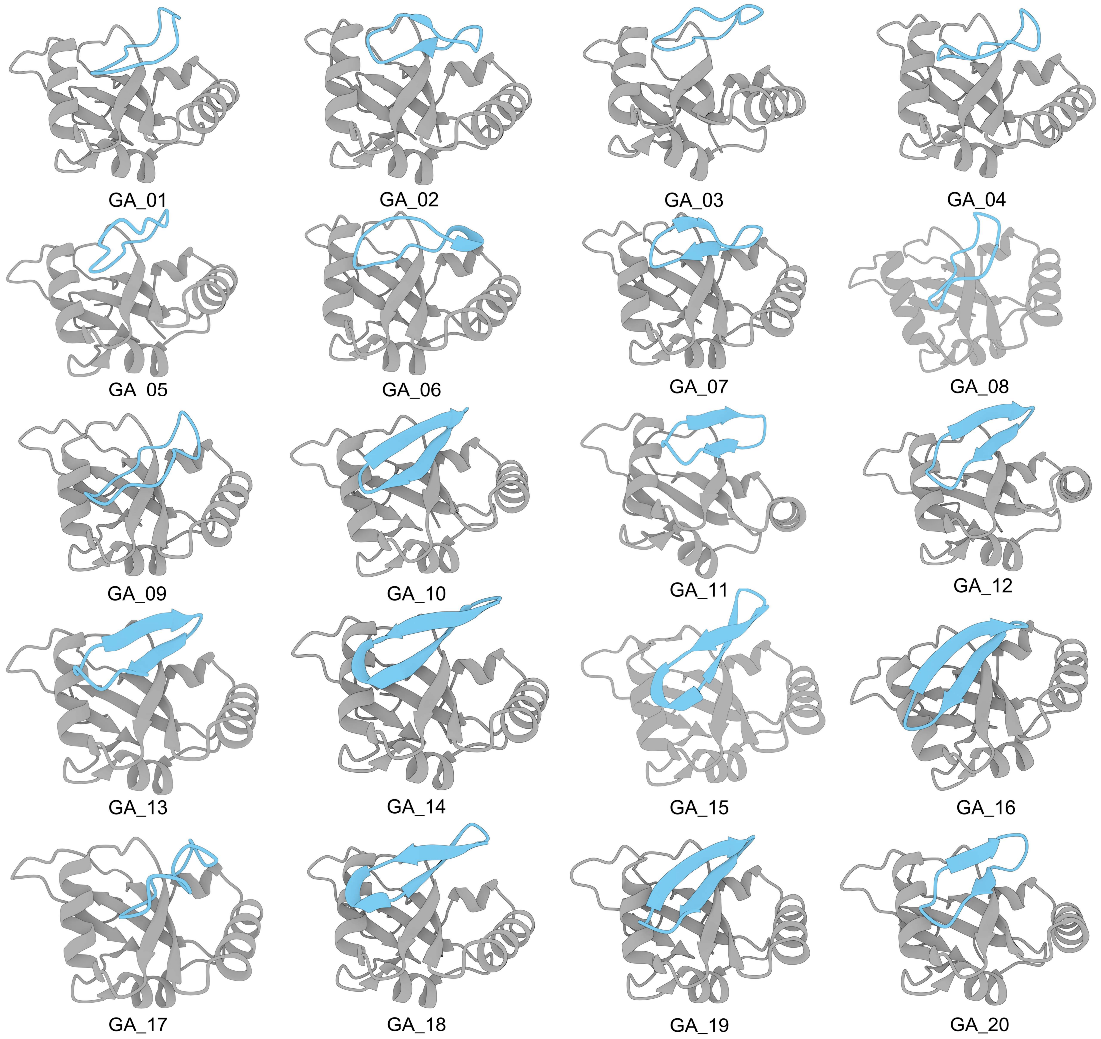

Fig.S7 Structural diversity of GABARAP-binding cyclic peptides designed by HFGuidedDesign

Section 8 Chemical Synthesis and Analytical Characterization of GABARAP-Targeting Cyclic Peptides

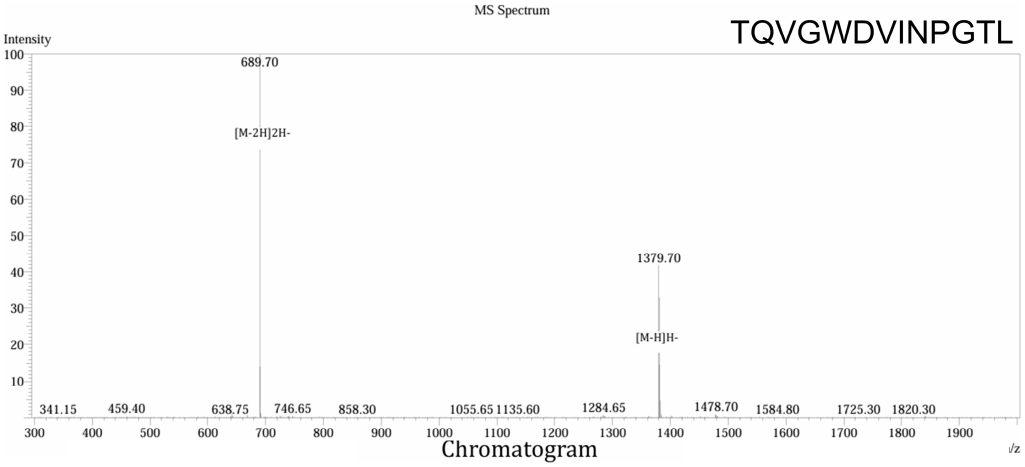

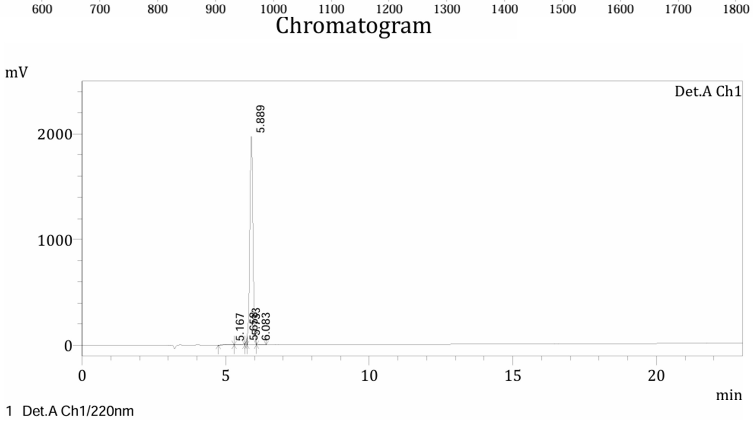

PeakTable

Detector A Ch1 220nm

Peak#	Ret. Time	Area	Height	Area %	Height %
1	5.167	45773	4152	0.303	0.203
2	5.658	61611	8917	0.408	0.436
3	5.733	154345	55882	1.022	2.730
4	5.889	14808093	1970012	98.090	96.252
5	6.083	26609	7763	0.176	0.379
Total		15096430	2046726	100.000	100.000

Fig.S8 Analytical HPLC and mass spectrometric characterization of the GABARAP-targeting cyclic peptide GA_05 designed by HFGuidedDesign.

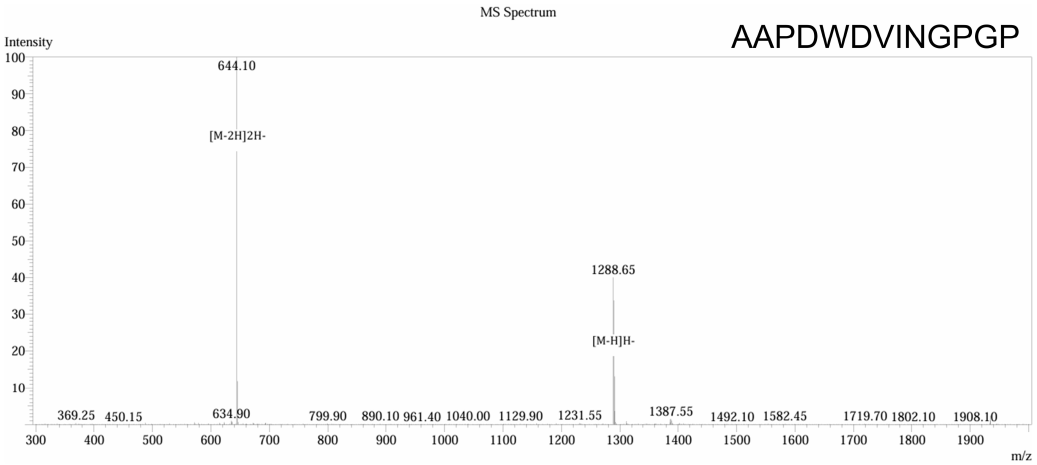

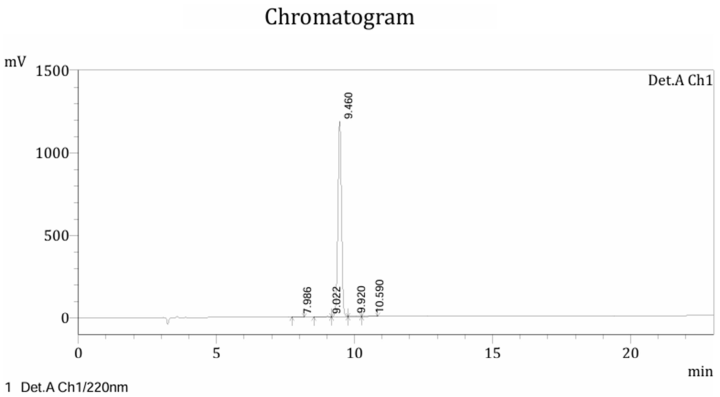

PeakTable

Detector A Ch1 220nm

Peak#	Ret. Time	Area	Height	Area %	Height %
1	7.986	8021	1034	0.074	0.086
2	9.022	14694	1594	0.136	0.133
3	9.460	10646640	1185289	98.396	99.063
4	9.920	69272	4243	0.640	0.355
5	10.590	81585	4339	0.754	0.363
Total		10820211	1196500	100.000	100.000

Fig.S9 Analytical HPLC and mass spectrometric characterization of the GABARAP-targeting cyclic peptide GA_01 esigned by HFGuidedDesign.

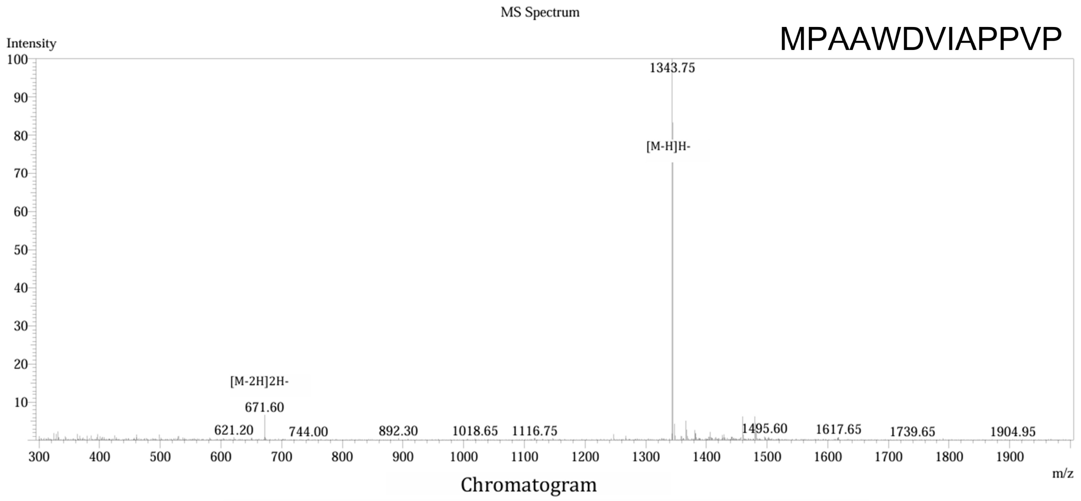

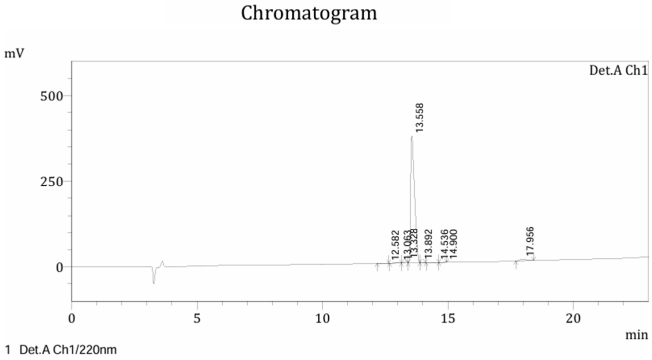

PeakTable

Detector A Ch1 220nm

Peak#	Ret. Time	Area	Height	Area %	Height %
1	12.582	2034	85	0.049	0.022
2	13.063	6296	444	0.151	0.116
3	13.328	36087	5133	0.864	1.341
4	13.558	4031812	369976	96.526	96.662
5	13.892	6818	1960	0.163	0.512
6	14.536	5272	286	0.126	0.075
7	14.900	1307	70	0.031	0.018
8	17.956	87307	4797	2.090	1.253
Total		4176933	382752	100.000	100.000

Fig.S10 Analytical HPLC and mass spectrometric characterization of the GABARAP-targeting cyclic peptide GA_08 esigned by HFGuidedDesign.

Section 9 Structural Stability and SPR Binding Validation of HFGuidedDesign-Designed Cyclic Peptides Targeting GABARAP

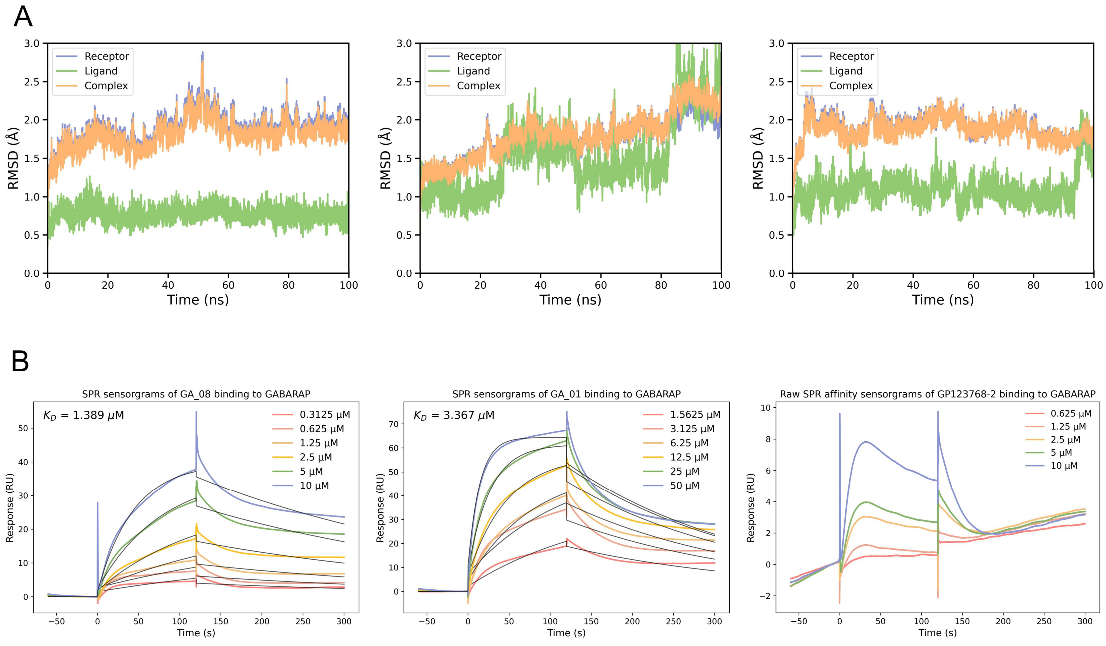

MPAAWDVLAPPVP　　AAPDWDVINGPGP　　TQVGWDVINPGTL

Fig.S11 Structural Stability and SPR Binding Validation of HFGuidedDesign-Designed Cyclic Peptides Targeting GABARAP.
